# Saudi mothers' experiences of caregiving within the care economy: a care diamond analysis

**DOI:** 10.3389/fpubh.2026.1897571

**Published:** 2026-10-07

**Authors:** Atheer AlSaif, Sama'a AlMubarak, Faisal Albagmi

**Affiliations:** Department of Public Health, College of Public Health, Imam Abdulrahman Bin Faisal University, Dammam, Saudi Arabia

**Keywords:** care diamond framework, care economy, mothers, qualitative research, Saudi Arabia, unpaid care work

## Abstract

**Objective:**

This study aims to explore Saudi mothers' caregiving experiences with particular attention to the activities, responsibilities, and emotional labor involved in maintaining family health and wellbeing. Using Razavi's care diamond framework, the study examines how these experiences are shaped by interactions across the family, state, market, and community sectors.

**Methods:**

An exploratory qualitative design was employed. Data were collected through six focus groups and four semi-structured individual interviews with 21 Saudi mothers recruited via purposive maximum variation sampling to capture diverse socioeconomic, caregiving, and geographical circumstances across Saudi Arabia. Sessions were conducted in Arabic, audio-recorded, and transcribed verbatim. Data were analyzed using deductive thematic analysis guided by the four sectors of the care diamond framework.

**Results:**

Six themes were identified: (1) mothers' caregiving work as activities and responsibilities; (2) caregiving, maternal meaning, and challenges to physical wellbeing; (3) gaps between state support and mothers' caregiving needs; (4) beyond the state: market and community role in caregiving; (5) the role of other caregivers in supporting mothers' caregiving; and (6) monetary valuation, recognition, and integration of unpaid care within the national economy. The care system was found to be income-stratified, shifting between semi-shared and near-unshared arrangements according to households' access to market-based services, with uneven support and limited coordination across sectors leaving caregiving concentrated within families and falling disproportionately on mothers.

**Conclusion:**

Saudi mothers function as de facto care coordinators within an income-stratified care system that shifts between semi-shared and near-unshared arrangements, where inconsistent, poorly coordinated support leaves unpaid caregiving labor unrecognized and concentrated within families, particularly on mothers. Policies should formally recognize, redistribute, and support unpaid care work in alignment with Vision 2030 goals.

## 1 Introduction

### 1.1 Background

Care sustains human life, promotes health and wellbeing across the life course, and underpins the productivity of formal economies. The care economy encompasses paid and unpaid activities that support the daily needs and wellbeing of individuals and households, with much of this work performed by women (1). Care extends beyond household tasks to include providing emotional support, caring for dependents such as children, older adults, and people with disabilities, and managing emotional and physical wellbeing (2, 3). The relational complexity of care explains why it's hard to measure or include the care economy in policy frameworks. The International Labor Organization (ILO) recognizes the care economy as vital to sustainable growth, a point reinforced by the COVID-19 pandemic, which highlighted the importance of care systems for social and economic wellbeing (4). Data from 64 countries show women perform 76.2% of unpaid care work globally, over triple men's share. This is equivalent to two billion people working 8 h daily without pay (5). If calculated at minimum wage rates, this unpaid labor would represent 9% of global GDP, approximately US$11 trillion (6, 7). If care were treated as an economy, it would be the third-largest after the United States and China, with a size over twice that of global agriculture (5, 8).

The care economy is among the fastest-growing formal sectors. In Australia, care is the largest and fastest-expanding part of the health and social support workforce (9). In the US, the formal care economy is valued at $648 billion annually (10). Care's growth impacts labor markets, investment, and social policy worldwide (6, 11). Governments worldwide adopt family-friendly policies to support mothers, recognizing the dual responsibility of employment and caregiving. Nordic countries use comprehensive welfare and gender equality to redistribute care (12, 13). Low- and middle-income countries find that paid leave and care programs increase maternal employment and family wellbeing (14).

A significant share of care work, especially that performed by women at home, remains unpaid, unrecognized, and largely excluded from national economic statistics and policy frameworks (6, 15). Feminist economists argue that unpaid care work operates as a hidden subsidy to formal economies supporting labor market participation and social reproduction, while excluded from economic recognition and accounting systems (16). Growing aging populations, declining fertility, and increased female labor participation have intensified demand for formal care. These shifts have also reframed care from a private family responsibility to a public concern with implications for gender equality, labor markets, social protection, and sustainable development (1, 6). Despite growing policy recognition, women's unpaid care work remains persistent, with women worldwide spending three to five times more hours than men (17, 18). Consequently, growing governmental recognition of care as a policy concern, reflected in international gender equality commitments, hasn't led to a proportionate redistribution of unpaid care, shifting from households to the state, market, or community. Mothers, in particular, continue to absorb care deficits, taking on the work that inadequate or inaccessible public services fail to provide (19, 20).

### 1.2 Motherhood, care work, and socioeconomic disadvantage

Across cultures and historical contexts, motherhood has been linked to caregiving, with mothers expected to shoulder primary responsibility for the physical, emotional, and developmental wellbeing (21, 22). Maternal care is a cultural expectation and a critical component of social reproduction and health, influencing children's nutrition, development, and psychological wellbeing (6). This responsibility is unevenly distributed. A mother's socioeconomic status, education, and access to services significantly shape her caregiving ability. Low-income mothers bear disproportionate caregiving demands due to limited access to social support and market alternatives (6, 14).

Empirical evidence from extensive cross-national studies further demonstrates the intersection of motherhood, care work, and socioeconomic disadvantage. The ILO used comparative statistical analysis of time-use and labor force data from 64 countries to measure the distribution of paid and unpaid care work (6). The findings revealed that women predominantly perform most unpaid care work worldwide. They also suggest that low-income women with limited access to services are more likely to reduce or leave paid employment due to caregiving duties. In the United States, a quasi-experimental study of paid family leave policies in California and New Jersey found that mothers with paid leave were less likely to leave the labor market after childbirth and more likely to stay employed for up to 5 years, especially among higher-educated mothers (14).

Systematic reviews focusing on low-income women further illustrate how socioeconomic disadvantage limits caregiving capacity. A systematic review of 34 qualitative studies across countries identified barriers such as transport difficulties, costs, discrimination, and limited family support, all of which restricted women's ability to care for themselves and their infants (23). Similarly, a systematic review of maternal health in low-income African countries found that economic hardship, cultural norms, and weak health systems collectively limit women's access to healthcare facilities and safe delivery options (24).

Beyond access to services, a systematic review of 19 studies involving approximately 70,310 participants found that unpaid labor has a negative impact on women's mental health, with less evident effects for men, emphasizing the psychological costs of gendered caregiving responsibilities (25).

Qualitative research on maternal mental load highlights the unseen cognitive and emotional effort in managing household and family responsibilities, linking it to increased stress and poorer mental health for mothers (26). A qualitative study across multiple countries India, Nepal, Rwanda, and Tanzania found that women balancing paid employment and unpaid care responsibilities often experience depletion, leading them to cut back on sleep, rest, and self-care to manage these competing demands (27). Similarly, comparative analysis of time use highlights unpaid care as the key factor explaining ongoing gender disparities in workforce participation, working hours, and job quality (28). However, much of this empirical literature either examines women or parents more broadly, rather than mothers specifically, or focuses on health access or labor market outcomes, rather than conceptualizing care as an economy. Much of this empirical literature is concentrated in Western contexts and broader MENA analyses; research situated specifically within Gulf countries, such as Saudi Arabia, remains scarce, and, to our knowledge, no studies explore how mothers' unpaid care work is organized, valued, and experienced within the care economy of this context. Specifically, there is limited understanding of how care responsibilities are managed and governed across the state, market, community, and family sectors, or how mothers value their unpaid caregiving compared to market-based estimates. The care diamond framework is well-suited to explore these questions (29). The main contribution of this study is empirical: it applies an established framework to a new context and level of analysis, where it has not been used before, offering a practice-based view of how mothers' unpaid care is organized, coordinated, and valued within Saudi Arabia's care economy (30).

### 1.3 Care economy in Saudi Arabia

The increasing recognition of the care economy, the persistent, gendered distribution of unpaid care, and rising demand for formal care services raise important questions about how care functions within specific national contexts, shaped by distinct cultural, institutional, and policy factors. Saudi Arabia serves as a particularly instructive example in this regard. As the largest country in the Gulf Cooperation Council (GCC) and one of the world's most rapidly transforming societies, Saudi Arabia is undergoing significant social and demographic change. At the same time, an ambitious national reform agenda under Vision 2030 operates alongside deeply rooted family-centered care norms that continue to shape caregiving arrangements across society (31). Although Vision 2030 reforms have expanded women's labor force participation and increased the availability of care-related services, care provision remains unevenly distributed across sectors. The state continues to play a dominant role, while contributions from the market and community remain more limited. Families, especially women, continue to be the backbone of daily care, despite the lack of formal policy recognition of their caregiving roles (30).

Within this broader transformation, Saudi Arabia's support systems remain limited and fragmented (30). Excessive caregiving demands compromise mothers' physical and mental wellbeing, with Saudi studies documenting reduced quality of life among caregivers of children with disabilities and moderate caregiver-related difficulties and perceived stress among parents of chronically ill children.

These structural support limitations are evident in families' daily lives in Saudi Arabia. Parents of children with disabilities engaged in health-promoting activities at lower rates, with higher education and better health status associated with greater engagement (32). This finding suggests that socioeconomic disadvantage may further compound the caregiving demands. Saudi mothers also face additional challenges, including emotional exhaustion, time poverty, and work-family conflict (33, 34). The ideology of intensive mothering, reinforced by cultural and religious norms, frames caregiving as a moral, gendered duty and pressures mothers to be endlessly selfless and constantly available (35).

Taken together, this evidence highlights a deeper problem of how unpaid care is recognized and valued in policy. Despite increasing global recognition of the care economy, Saudi policy still affords limited formal acknowledgment to the market, community, and, particularly, the family sector. While the state remains the dominant provider of care services, the unpaid contributions of families, particularly mothers as primary caregivers, are largely absent from official policy narratives, administrative records, and statistical data. This limits understanding of the scope and gendered distribution of unpaid care work and may constrain progress toward the goals of Saudi Vision 2030 (30). This gap forms the foundation of the present study, which explores Saudi mothers' caregiving experiences using Razavi's Care Diamond framework (29).

### 1.4 The care diamond framework

The care diamond, developed by Razavi, is a comprehensive analytical framework that conceptualizes care responsibilities as distributed across four interdependent sectors: the family, the state, the market, and the community (29). Unlike earlier binary frameworks that classified care as either public or private (12, 36), paid or unpaid (15), the care diamond acknowledges that care is produced through the simultaneous, often unequal interaction among all four sectors. It also recognizes that this interaction varies greatly across different social, political, and economic contexts (12, 29). When a sector withdraws or underperforms, responsibility for care shifts elsewhere. It often falls to the family and, within families, disproportionately to women (20, 29).

The framework has since been applied comparatively across diverse welfare regimes. An empirical policy analysis of Serbia mapped the distribution of childcare and eldercare across the four sectors of the care diamond (37). Their findings showed that the informal family sector was dominant in both domains, while the voluntary sector was entirely absent from childcare, indicating that incomplete institutional support concentrates the residual care responsibilities within families and particularly mothers. Alternatively, Rogero-García in a comparative quantitative study drawing on United Nations and European Commission demographic data across six world regions, extended the care diamond framework to distinguish between shared, semi-shared, and unshared care systems (38). The findings demonstrated that regions with weaker formal-sector involvement, particularly in Africa and Latin America, have disproportionate levels of unpaid care work, with women undertaking the majority of this work. The care diamond framework was applied to childcare and eldercare in Japan, showing that Long-Term Care Insurance reforms reduced the family's share of care responsibilities (39). Kofman & Raghuram extended the framework to migration and transnational care, showing how labor migration from the Global South reshapes care arrangements across the four care diamond sectors and can create care gaps in origin households (40).

In the Saudi context, the care diamond was applied to examine the interactions between state, market, family, and community across the domains of health, education, and social care, finding that the state sector dominates, while family-based care remains structurally invisible and policy-unrecognized (30). The present study expands this foundation by shifting the analytical lens from institutional policy texts to the lived experiences of Saudi mothers, examining how the four sectors of the care diamond interact in daily caregiving activities and identifying where the remaining care responsibilities ultimately fall. When describing how responsibility is distributed, the analysis also references Rogero-García's classification of care systems as shared, semi-shared, or unshared (38). Here, this classification serves as a descriptive tool to summarize the configuration revealed by the care diamond.

### 1.5 Research aim and objectives

The objective of this study is to explore how Saudi mothers understand and experience their caregiving roles, with particular attention to the activities, responsibilities, and emotional labor involved in maintaining family health and wellbeing. Using Razavi's care diamond as a theoretical framework, the study explores how mothers' caregiving practices are shaped through their interactions with the family, state, market, and community sectors.

### 1.6 Significance of the study

This study makes a significant contribution to care economy research in Saudi Arabia by providing an in-depth qualitative account of caregiving within the family sector. By centering the voices of Saudi mothers through focus groups and individual interviews, the study moves beyond policy-level analysis to capture the lived realities of everyday caregiving. The findings show that caregiving involves continuous, multidimensional work, including physical, emotional, and cognitive coordination, with mothers remaining central caregivers even with support from the state, market, community, or family. The study further documents the structural constraints, institutional gaps, and social expectations that shape mothers' caregiving, influencing their responsibilities and wellbeing. This research also contributes to the global care economy literature, which is mostly theoretical or policy-focused, by providing empirical evidence from mothers' lived experiences that strengthens the shift from theory to practice-informed understanding. It emphasizes Saudi Arabia's unique Gulf context, largely absent from the care economy literature. Most research focuses on Western welfare states or broad MENA analyses, overlooking Gulf-specific institutional, cultural, and policy aspects. By situating the study in Saudi Arabia, the findings provide a contextually grounded perspective on care provision that goes beyond Western assumptions. The study also offers a transferable model for analyzing care economies in comparable societies, such as other Gulf Cooperation Council GCC countries, where rapid social transformation, family norms, and limited formal care infrastructure intersect. The study further broadens the application of Razavi's care diamond framework by adopting mothers' perspectives as the primary actors within the family sector, rather than focusing on policy analysis or institutional mapping. This approach reveals how care systems function in practice beyond policy design, identifies the gaps absorbed by informal labor, and highlights effective intervention points to reduce the caregiving demands on women. Finally, the study contributes to the literature on women's and mothers' wellbeing by examining how caregiving affects their physical, emotional, and social health, an area largely overlooked in MENA research, which tends to focus on employment, education, or healthcare access. It enhances understanding of gendered health and welfare in Saudi Arabia and similar contexts and offers original empirical evidence to inform the re-examination of care arrangements and support the development of policies that are equitable, gender-responsive, and aligned with national development goals.

## 2 Methodology

### 2.1 Research design

This study employed an exploratory qualitative design to examine how Saudi mothers carry out caregiving activities and responsibilities in relation to their families' health and wellbeing, guided by the care diamond framework. Qualitative research explores social phenomena in depth by analyzing non-numeric data, focusing on meanings, experiences, and interpretations within specific social, cultural, and economic contexts (41). This approach enabled a detailed investigation of Saudi mothers' everyday experiences of “living through situations” related to family health and wellbeing. Using this design allowed me to conduct a detailed exploration of their caregiving practices within the family context and their experiences of navigating and interacting with state, market, and community care sectors in everyday caregiving. Its context-sensitive nature enabled an in-depth understanding of caregiving in the Saudi setting, where social norms, institutional arrangements, and family structures shape care responsibilities (42). In addition, the flexible design allowed the incorporation of insights that emerged during data collection and analysis, including aspects of caregiving not fully addressed in the existing literature (43). By facilitating the identification of recurring themes and patterns across mothers lived caregiving experiences, the qualitative design provided a structured and interpretative understanding of unpaid caregiving within Saudi households, situated within the broader arrangements of family, state, market, and community care.

### 2.2 Sampling strategy and participants

Participants were recruited through purposive sampling, also called purposeful or judgemental sampling, a non-probability sampling strategy in which researchers intentionally select participants most likely to provide rich, relevant, and useful information about the phenomenon of interest (44, 45). Maximum variation purposive sampling was used to capture a wide range of caregiving experiences by intentionally including mothers who differed in educational level, employment status, household income, number of children, access to domestic help, and region of residence, enabling caregiving experiences to be examined across diverse social, economic, and geographic contexts. Maximum variation sampling seeks out heterogeneous cases along key dimensions in order to document important common patterns that cut across variation as well as meaningful differences (45).

Accordingly, Saudi mothers who were primary caregivers for their families were recruited to capture a diverse range of caregiving experiences. Participants were recruited from the central, eastern, western, southern, and northern regions of Saudi Arabia and included both married and divorced mothers. The final sample included 21 Saudi mothers, of whom 17 participated in six focus groups and four in individual interviews. Most participants were in their thirties and held at least a bachelor's degree. The majority lived in the Eastern Province of Saudi Arabia, with additional participants from the central, western, southern, and northern regions. Most mothers were employed, mainly in full-time positions, and the largest proportion of households reported monthly incomes of 10,000–14,999 SAR. In terms of caregiving, most mothers cared for two to four children of mixed ages, almost all identified themselves as the primary caregiver, and the majority reported routine childcare responsibilities alongside regular access to paid domestic help. Detailed demographic and caregiving characteristics are presented in Tables 1, 2. This non-probabilistic approach enabled me to recruit participants who were most likely to provide rich insights into how caregiving activities and responsibilities are organized and experienced within the family, and how mothers engage with state, market, and community-based care sectors.

**Table 1:** Demographic characteristics of research participants (*N* = 21).

Characteristic	Number	Percentage %
Age (years)		
20–29	2	9.5%
30–39	14	66.6%
40–49	4	19%
≥50	1	4.7%
Marital status		
Married	18	85.7%
Divorced	3	14%
Education		
High school	0	0
Diploma	0	0
Bachelor's degree	15	71.4%
Postgraduate degree	6	28.5%
Employment status		
Full-time	15	71.4%
Part-time	1	4.7%
Unemployed	5	23.8%
Region of residence		
Eastern	16	76.2%
Central	1	4.7%
Western	1	4.7%
Southern	2	9.5%
Northern	1	4.7%
Household income (SAR)		
< 5,000	0	0
5,000–9,999	3	33.3%
10,000–14,999	12	57%
≥15,000	6	30%

**Table 2:** Caregiving-related characteristics of research participants (*N* = 21).

Characteristic	Number	Percentage %
Number of children		
1	2	9.5%
2–4	17	81%
≥5	2	9.5%
Age of children		
Only young children (infant/preschool)	1	4.7%
Mixed ages (young and school-aged/adolescent)	20	95%
Caregiving responsibilities		
Routine childcare	19	90.5%
Child with disability/special needs	1	4.7%
Older adults dependent	1	4.7%
Primary caregiver		
Mother	21	100%
Others	0	0
Access to domestic help		
No domestic help	2	9.5%
Irregular/occasional domestic help	1	4.7%
Regular domestic help (nanny/domestic worker/driver)	18	86%

I used an online screening survey to identify and select eligible participants for the focus groups and individual interviews, which also served as their initial consent, distributed prior to data collection. I shared the survey via public social media platforms, enabling mothers from various regions and circumstances to self-select without any prior contact. I did not know any of the participants personally before recruitment. The survey aimed to recruit a diverse sample of Saudi mothers and was organized into sections on demographics, family composition and socioeconomic background. It also included items on caregiving responsibilities, the types of care provided, and the approximate number of hours spent on caregiving each day. The final section asked about willingness to participate and preferred mode of participation: 1-attending in-person focus groups, 2- joining via Zoom, or 3-opting for a one-to-one interview. Providing flexible participation options was a deliberate strategy to reduce barriers to participation and to ensure that mothers with different circumstances such as geographic distance, caregiving demands, or personal preferences could participate in the study. Recruitment involved a second stage of chain-referral (snowball) sampling, whereby mothers who completed the screening survey and agreed to participate shared the survey link with other eligible mothers in their social networks, expanding the pool of potential participants (46). All referred mothers completed the same screening survey and met the same criteria as those recruited directly, ensuring consistent selection procedures across recruitment routes. Eligible participants were Saudi mothers with active caregiving responsibilities for at least one child.

Focus groups are most effective when participants share some commonalities relevant to the research topic (47). Accordingly, this study employed largely homogeneous focus groups by grouping mothers with broadly similar employment status, socioeconomic position, and caregiving intensity. This grouping was designed to facilitate open discussion and minimize potential discomfort or power imbalances when addressing sensitive topics such as income, domestic help, and employment. For example, working mothers and stay-at-home mothers were typically placed in separate focus groups because mixing them could hinder open discussion due to concerns about judgment, comparison, or stigma. Likewise, mothers from various economic backgrounds were grouped with others of similar socioeconomic status, acknowledging that topics such as household income, access to private services, and paid care are sensitive and may reduce participation in mixed-socioeconomic status groups. Caregiving intensity was also a key consideration in the sampling and group formation process. Although focus groups were internally homogeneous, diversity was ensured across the overall sample through the maximum variation strategy described above.

### 2.3 Data collection

The researcher collected data using focus groups and semi-structured individual interviews with Saudi mothers. The researcher adopted this multi-method qualitative approach to capture both shared and individual caregiving experiences and to maximize the depth and richness of the data. Both methods followed the same semi-structured interview protocol and were conducted during the same data collection period, with sessions of similar length and held in comparable settings. In total, I conducted six focus groups and four individual interviews.

### 2.4 Focus group

Focus groups are a method of data collection that is deceptively straightforward, involving assembling a small group of participants, usually between 2 and 12 people, to engage in an informal discussion around a specific topic of choice. A focus group facilitator guides the conversation using a list of open-ended questions to ensure group members' engagement (48, 49). I used focus groups as a primary data collection method because they are particularly effective for exploring shared experiences, social norms, and collective meanings surrounding everyday practices such as caregiving. The interactive nature of focus groups encourages reflection, comparison, and elaboration of experiences that may not emerge in individual interviews (47). In these focus groups, I met with participants to discuss caregiving activities, conducting sessions both in person and online via Zoom, especially for participants living in remote areas. Focus groups vary in size and duration depending on study objectives, the topic under discussion, its sensitivity and complexity, the desired balance between breadth and depth of coverage, and the population, and there are no fixed rules about how many to include in a focus group (47). Accordingly, the size of focus groups was intentionally kept small (two to four participants per group) to allow sufficient time for each participant to contribute and facilitate deeper discussion of caregiving experiences. Smaller groups were particularly appropriate given the personal and sensitive nature of caregiving, family health, and emotional labor (50). Group composition reflected participants' preferences and scheduling constraints, resulting in two of the six groups having only two participants. These sessions are more accurately described as dyads, where two participants and the moderator engage in a triangular discussion, sitting between an individual interview and a traditional focus group (51). Such small formats are considered suitable when topics are sensitive, when each participant needs to share substantial personal insights, and when recruiting the population is challenging (52). These sessions followed the same protocol as larger groups, encouraging participants to respond to each other's accounts to maintain peer interaction.

I conducted the focus groups using a semi-structured interview protocol that I developed for this study, guided by the care diamond framework (Appendix A). The protocol was organized into thematic sections covering daily caregiving roles and responsibilities, health and emotional wellbeing, support systems and sectoral interactions, financial value and unpaid care, the role of domestic workers, personal impact and wellbeing, and recommendations and reflections. Within these sections, I included open-ended questions such as “Can you walk us through a typical day in your life as a caregiver?,” “How do you manage your family's health needs?,” “Who supports you in your caregiving role?,” and “If you had to hire someone to replace you for your daily caregiving tasks, what job titles best match your duties?” to encourage detailed and reflective responses. The semi-structured interview protocol was originally developed in English and then translated into Arabic; all focus group and individual interview questions were administered in Arabic to match participants' native language (Appendix B). To ensure translation accuracy and conceptual equivalence, I reviewed the Arabic version with a bilingual qualitative researcher and refined wording where needed to preserve the intended meaning and cultural appropriateness. I developed these questions iteratively by mapping the study aim and the four sectors of the care diamond (family, state, market, and community) onto key areas of inquiry and refining the wording for clarity, cultural appropriateness, and openness.

To establish content and face validity, I asked three experts in public health and qualitative research to review the protocol for relevance to the study aim and comprehensive coverage of the care diamond sectors, and I revised the questions based on their feedback by simplifying language and reordering sections to follow the flow of a typical caregiving day (53). I then pilot-tested the interview protocol with two Saudi mothers who met the inclusion criteria but were not included in the main study, observing how they understood the questions and the richness of their responses. The pilot highlighted the need to clarify certain terms, adjust the order of some questions, and add prompts for emotional and financial aspects of caregiving, and I incorporated these changes before using the final version of the semi-structured protocol in all focus groups (54, 55).

### 2.5 Individual interviews

I conducted semi-structured individual interviews as a complementary method with mothers who were unable or preferred not to join a focus group, due to scheduling constraints, caregiving responsibilities, or comfort with discussing personal experiences one-to-one. As with the focus groups, these interviews followed the same semi-structured protocol, were conducted during the same data collection period, and used identical procedures for setting, audio recording, language, transcription, and translation.

I recruited participants and collected data concurrently for both focus groups and individual interviews between 19 June and 28 September 2025, during which six focus groups and four individual interviews were conducted. The focus groups included 17 participants, with two to four mothers per group, while individual interviews were conducted to accommodate participants' preferences and remote locations. Each focus group session and interview lasted approximately 90 min. In-person sessions were held in private meeting rooms within public venues chosen for their comfort, accessibility, and suitability for sensitive discussions.

I obtained informed consent from all participants before data collection. The consent form was available in both English and Arabic, but participants reviewed and signed the Arabic version, which is provided in Appendix C, while the English version is presented in Appendix D for reference. For focus groups and individual interviews conducted in person, participants signed a written consent form at the start of the session. For online interviews and focus groups held via Zoom, I emailed participants an electronic version of the consent form in advance and obtained their electronic signatures before the meeting. I initially contacted mothers using the contact details they provided in the screening survey and followed up by email and phone to share the participant information sheet, confirm eligibility, arrange convenient times, and answer any questions about the study. The screening survey included an information statement about the study and a question asking whether mothers were willing to be contacted for participation, which served as an initial expression of interest; formal written or electronic consent was then obtained as described above. Throughout data collection, the researcher repeatedly assured participants of the anonymity and confidentiality of their responses. All sessions were audio-recorded using a digital recorder for in-person meetings and Zoom's recording function for online sessions, with participants' consent.

### 2.6 The caregiving valuation exercise

Both focus groups and individual interviews included a caregiving valuation exercise, included as a specific part of the semi-structured protocol on the financial value of unpaid care (Appendix A). Mothers were asked to identify their main daily caregiving and household tasks, estimate the time dedicated to each, and specify the paid job titles that would match these tasks if someone else were hired to do them. To capture both perceived and market-based dimensions of care, this study employed a dual valuation strategy. First, mothers were asked to assign monetary values to their unpaid care activities based on their perceived physical, emotional, and cognitive effort rather than prevailing market wages. These effort-based valuations were summed across activities for each participant and averaged across the 21 mothers. Second, the researcher used the replacement-cost method, applying locally relevant market wages for comparable paid-care roles that participants had identified (e.g., nanny, cook, cleaner, nurse, and tutor) (56). This produced an external benchmark against which mothers' subjective valuations could be compared with the formal economic pricing of care labor. The exercise functioned primarily as a qualitative tool rather than providing quantitative data. Its goal was to encourage reflection and discussion, helping mothers express the effort, continuity, and responsibility involved in caregiving and relate this work to the paid economy. The monetary figures presented in the findings are thus regarded as qualitative summaries of participants' judgments; no statistical or inferential claims are made, nor is there an intent to generalize beyond the sample. The comparison between effort-based and replacement-cost valuations serves analytically to explore how mothers view caregiving in relation to market pricing and what aspects such pricing may overlook.

### 2.7 Translation and transcription

All focus group discussions and individual interviews were conducted in Arabic, the participants' native language, and were audio-recorded with their consent. The researcher transcribed the recordings verbatim to maintain the original wording, meanings, and expressions of the participants (57). The transcription process lasted 1 month and involved repeatedly listening to the recordings to ensure both accuracy and completeness. Conducting the transcription personally enabled deeper engagement with the data and facilitated early familiarization with the participants' caregiving experiences. As the researcher is bilingual and fluent in both Arabic and English, she conducted the translation herself (58–60). Importantly, full transcripts were retained in Arabic for analysis, and translation into English was limited to selected quotations used in the written narrative. This approach helped maintain the authenticity of participants' voices while minimizing the risk of meaning loss that can occur when translating full data (61). Translating only the quotations presented in the findings is consistent with best practice in cross-language qualitative research, as it keeps analysis grounded in the original language while ensuring accessibility for an English-speaking audience (58, 62). Offering both native and translated quotes can further boost the symbolic and conceptual value of qualitative research. Original Arabic quotations and their corresponding English translations are provided in Appendix E to enhance transparency and preserve meaning across languages.

### 2.8 Data analysis

In this study, a deductive approach to thematic analysis was adopted. The data were analyzed using thematic analysis, a systematic approach for identifying, analyzing, and reporting patterns in qualitative data (63, 64). Thematic analysis is particularly well-suited to studies that aim to provide a rich, detailed, and nuanced account of participants' perspectives (63). Thus, thematic analysis was employed to explore how Saudi mothers interpret and attribute meaning to their caregiving experiences regarding their families' health and wellbeing within their sociocultural contexts. The analysis was guided by the care diamond framework, which conceptualizes care provision across four key sectors: the state, the market, the family, and the community (29). This framework was selected because it aligns with the study's aim of examining caregiving responsibilities within broader social, economic, and institutional structures. The care diamond provided an organizing frame for the initial coding structure, while still allowing the data to inform the refinement and nuance of themes within each sector.

The analytic process followed Braun & Clarke six-phase structure (63). The first phase focused on familiarization with the data through repeated reading of transcripts and listening to audio recordings. During this stage, reflexive notes were taken to capture early observations of mothers' caregiving experiences. In the second phase, systematic coding was conducted using NVivo 15 software (Lumivero, LLC, Denver, CO, USA), during which meaningful data segments were labeled and mapped deductively onto the four components of the care diamond framework (65). In the third phase, codes were organized into preliminary themes, while in the fourth phase, these themes were reviewed for coherence, consistency, and alignment with both the data and the conceptual framework. The fifth phase focused on refining and defining the final themes, ensuring conceptual clarity and distinctiveness across thematic categories. In the final sixth phase, the themes were synthesized into a coherent analytic narrative that captured patterns across participants' experiences.

### 2.9 Trustworthiness

To ensure the trustworthiness of the analysis, this study drew on the criteria for credibility outlined by Lincoln et al. (66). First, credibility was strengthened through methodological triangulation, using multiple qualitative sources, including focus groups, individual interviews, and insights drawn from a prior qualitative study using document analysis that I conducted and published (30). In that study, I analyzed Saudi policy documents and official webpages on the care economy, which showed that family caregiving, particularly mothers' unpaid roles, is only minimally and indirectly addressed in official policies, despite the family's central position in the care diamond framework. The focus groups and interviews reported in the current study revealed detailed accounts of intensive, everyday caregiving that are largely invisible in these policy texts. Triangulating the document-analysis findings with mothers' narratives strengthened credibility by demonstrating a clear gap between how care is framed in policy discourse and how it is experienced in practice, based on evidence from both documentary and interview data.

Second, credibility was further supported through member checking (respondent validation), in which selected participants were invited to review and reflect on the preliminary findings and emerging themes. This process gave participants the opportunity to confirm that the interpretations accurately reflected their experiences and meanings, reduce misinterpretations and identify potential researcher bias or misunderstanding (67). Third, Adequate engagement with the data also contributed to trustworthiness. Data collection and analysis proceeded iteratively until thematic saturation was reached. Consistent with Lincoln and Guba's recommendation to continue sampling until redundancy or saturation is achieved, I continued collecting data until no substantially new themes or insights emerged (66). Saturation was identified after analyzing six focus groups and four individual interviews, at which point additional data largely repeated existing patterns rather than adding new conceptual categories.

Reflexivity was maintained throughout the research process. As a Saudi mother-researcher with a background in public health and the care economy, I possessed contextual familiarity with caregiving practices and policy environments in Saudi Arabia. While this positionality supported informed and context-sensitive interpretation, I actively used reflexivity as a strategy to minimize potential bias. I kept a reflexive journal in which I documented my assumptions, emotional responses, and methodological decisions after each focus group and interview, and I returned to these notes during analysis to question how my position might be shaping interpretations. I also continually reviewed my analytic decisions and revisited the coding. I checked these decisions against the data and the care diamond framework to keep interpretations grounded in participants' accounts rather than my prior assumptions. I used NVivo software for coding and analysis, regularly reviewing and discussing coding decisions and emerging themes with the research supervisor to ensure consensus and explore alternative interpretations. Collectively, these strategies enhanced the study's credibility and overall trustworthiness.

### 2.10 Ethics and consent

Informed written consent was obtained from each participant prior to the focus group sessions and individual interviews, ensuring confidentiality and anonymity. The study was approved by the Ethics Committee of Imam Abdulrahman bin Faisal University (IRB-2024-03-629) on September 18, 2024.

## 3 Results

Thematic analysis of focus group and interview data from Saudi mothers identified six interrelated themes that capture the intensity and multidimensional nature of caregiving, how mothers navigate and compensate for gaps in support, and the broader care arrangements that shape caregiving in practice. The themes are: (1) mothers' caregiving work as activities and responsibilities; (2) caregiving, maternal meaning, and challenges to physical wellbeing; (3) gaps between state support and mothers' caregiving needs; (4) the role of the market and community in caregiving beyond the state; (5) the role of other caregivers in supporting mothers' caregiving; and (6) monetary valuation, recognition, and integration of unpaid care within the national economy. Collectively, the findings reveal an income-stratified care system in which arrangements shift between semi-shared and near-unshared configurations according to households' access to market-based services, with uneven support and limited coordination across sectors leaving caregiving concentrated within families and falling disproportionately on mothers.

### 3.1 Theme 1: Mothers' caregiving work: activities and responsibilities

Participants described caregiving as demanding, multidimensional work that structured their daily lives. Across regions and family circumstances, mothers framed caregiving as a combination of physical tasks, emotional and cognitive labor, and responsibility for coordinating children's education, health, and daily routines. This theme focused on the nature of caregiving work and the conditions that shape it, encompassing three interrelated subthemes: (1.1) the physical and domestic labor of caregiving; (1.2) cognitive coordination, emotional labor, and health navigation; and (1.3) educational and economic factors shaping caregiving. This theme primarily reflects the family sector of the care diamond, where mothers described themselves as central to the organization and delivery of everyday care.

#### 3.1.1 The physical and domestic labor of caregiving

Participants described caregiving as continuous, time-extensive daily labor involving managing morning and evening routines, preparing children for school, organizing household tasks, and coordinating multiple schedules under significant time pressure during peak hours. Working mothers emphasized that employment added an extra layer of organization to this workload, particularly in the mornings when they had to prepare themselves and their children, manage school drop-offs, and arrive at work on time. As one working mother from the Western region explained,

“*I have to get myself ready and prepare my children at the same time. When you have more than two children in different schools, you have to wake up very early, and the whole situation becomes difficult to manage*” (Participant 3).

These responses were common among the 15 full-time workers, who saw caregiving as ongoing throughout the workday rather than starting after work. Instead of being done in free time after work, caregiving duties overlapped with employment hours within the same limited morning and evening periods. As a result, fulfilling one set of responsibilities often meant sacrificing the other.

Mothers emphasized that alongside child-focused care, a continuous cycle of household chores such as preparing meals, cleaning, doing laundry, and restocking supplies was integral to caregiving. These chores were rarely seen as separate activities but as ongoing work woven throughout the day, often overlapping with childcare tasks. Mothers often shifted between feeding, tidying, and supervising within the same timeframe. Mothers with regular paid domestic help described directing and overseeing this work rather than performing it all themselves.

#### 3.1.2 Cognitive coordination, emotional labor, and health navigation

Beyond physical tasks, mothers consistently highlighted the cognitive and emotional aspects of caregiving, particularly the ongoing planning, coordination, and decision-making required to manage family life. Participants described how caregiving involved constant attention to multiple responsibilities, often simultaneously. One mother explained,

“*The most demanding part is coordination; deciding what's for lunch, who will take the kids to football practice, and what household items need to be restocked. They seem small, but the mental load and constant planning are draining*” (Focus group 1).

Mothers described emotional labor as a core component of caregiving, involving ongoing reassurance, guidance, and emotional regulation, indicating that caregiving extended beyond practical tasks to sustained emotional engagement.

Mothers' discussions revealed shared perceptions that caregiving is primarily viewed as a mother's responsibility, with strong societal expectations that mothers should manage care independently. Many mothers described feeling judged when seeking help, noting that reliance on support was often interpreted as maternal inadequacy, while fathers were held to lower expectations. As one participant explained,

“*Any shortcomings from husbands are forgiven, while mothers are criticized for the smallest slip*” (Focus group 1).

Health-related caregiving was a major focus in mothers' accounts, partly because the study framed caregiving as a form of labor that supports family health, and partly because health needs involved the most complex coordination among providers and institutions. Within this context, mothers consistently described healthcare navigation as resting almost entirely on them, regardless of employment status or family arrangements. They described being responsible for organizing and managing children's healthcare, using phrases such as “*the mother bears full responsibility*” and “*everything related to health at home is my responsibility*.” Health-related caregiving tasks included arranging medical appointments, attending follow-ups, monitoring symptoms, administering medication, and maintaining vaccination and check-up records. One mother explained that

“*The periods requiring the greatest emotional and cognitive effort were when children were ill, since I naturally tend to worry a lot*” (Participant 2).

Differences emerged in the organization of health-related caregiving, particularly for children with disabilities, where care involved multiple unconnected providers and services. Mothers described having to coordinate appointments with different specialists and hospitals, resulting in more time-intensive, demanding caregiving routines. A working mother caring for a child with a disability explained:

“*I have to take regular excuses from work because of the continuous follow-up and medical check-ups for my child. Moving between different hospitals and doctors takes a lot of time and effort and causes me difficulties at work*” (Focus group 4).

In contrast, mothers of children without chronic or complex conditions described health-related caregiving as a routine task, centered around preventive care and occasional illness management.

#### 3.1.3 Educational and economic factors shaping caregiving

Mothers agreed that education shaped caregiving but differed in whether it eased or intensified responsibilities. These differences emerged across participants with varying educational and professional backgrounds. Mothers with higher levels of education or professional training more frequently described caregiving as intensified by heightened expectations for children's development, academic achievement, and enrichment activities. In contrast, mothers who placed greater emphasis on practical experience rather than formal qualifications described education as supporting caregiving through improved understanding of children's learning and health needs. For example, one participant working in a health-related field described how her professional knowledge enabled her to identify a health concern in her child early. Mothers with medical backgrounds reported that professional knowledge heightened vigilance in monitoring children's health, sometimes leading to increased concern over minor symptoms (Participant 4). At the same time, several highly educated mothers emphasized that this heightened awareness translated into greater involvement, supervision, and coordination, increasing the scope of caregiving work. As one mother explained,

“*As an educated mother, I know extracurricular activities benefit my children, so I make sure to enroll them in sports, language classes, and skills such as coding. Organizing all these activities takes a lot of energy and effort*” (Focus group 1).

Conversely, a divergent perspective emerged in one focus group, where a highly educated mother questioned the relevance of formal education in shaping caregiving capacity. Drawing on her observations of other mothers in her social environment, she argued that caregiving competence depended more on personal characteristics such as attentiveness, patience, and commitment than on educational attainment alone. As she stated,

“*The educational level does not make a difference in providing care… it depends on the mother's personality*” (Focus group 2).

This negative case highlights that education was not consistently perceived as a defining factor in caregiving quality. Instead, it suggests that some highly educated mothers interpret caregiving through a relational and experiential lens, where personal dispositions and daily practices are viewed as more influential than formal qualifications. This perspective shows that the role of education in caregiving is not uniform but experienced in different and sometimes conflicting ways.

The analysis found broad consensus among participants that economic status significantly shapes caregiving, though mothers disagreed on whether its effects were primarily positive or negative. Many participants described higher income as facilitating caregiving by enabling access to private schooling, extracurricular activities and domestic help. Financial stability was seen as enabling mothers to access more resources and provide better-quality care for their children. As one mother explained,

“*Because of my good financial situation, I was able to enroll with a specialist family consultant and acquire skills that helped me care for my children services I would not have been able to access without sufficient income*” (Focus group 4).

At the same time, some mothers argued that higher economic status intensified caregiving demands by raising expectations. One participant explained that

“*A higher economic status can actually add to the family's burdens, as expectations and demands rise, increasing psychological pressure on mothers*” (Focus group 1).

This pressure was particularly evident among middle-class families, who described striving to maintain educational and lifestyle standards associated with higher socioeconomic groups, leading to financial strain from school fees and extracurricular activities. Some mothers described sacrificing personal needs or limiting expenses to prioritize their children's education. This complexity was especially pronounced for mothers who were primary providers. A divorced mother from the Western region explained that financial independence was essential for meeting all household and childcare expenses, and that this stability enabled her to sustain caregiving responsibilities on her own (Participant 3). These findings illustrate that economic resources both support and complicate mothers' caregiving responsibilities. Caregiving also shaped mothers' employment and educational trajectories. Many explained that caregiving responsibilities required them to delay, modify, or abandon professional or academic goals, such as choosing jobs with fewer hours and less financial return, postponing postgraduate studies, or limiting professional development opportunities. Mothers frequently described prioritizing caregiving over career advancement, particularly during periods of intensive childcare:

“*I couldn't study abroad because I prioritized caring for my children over my academic advancement*” (Focus group 2).

Together, these accounts portray caregiving as an intensive, structured form of daily labor, encompassing physical tasks, cognitive coordination, emotional engagement, and responsibility for children's health, carried out continuously across the day and shaped by mothers' educational and economic circumstances.

### 3.2 Theme 2: Caregiving, maternal meaning, and challenges to physical wellbeing

Mothers described caregiving as an experience that profoundly shaped their physical wellbeing and sense of self. Caregiving was consistently portrayed as physically demanding and time-intensive, affecting sleep, health, and opportunities for rest and self-care. At the same time, mothers articulated strong sources of meaning, fulfillment, and pride derived from caregiving, highlighting its dual role as both a source of strain and personal significance. Situated primarily within the family sector of the care diamond, this theme highlights three interrelated aspects of mothers' experiences: (2.1) physical exhaustion and health impacts; (2.2) loss of personal time; and (2.3) the positive meaning and fulfillment derived from caregiving.

#### 3.2.1 Physical exhaustion and health impacts

Mothers consistently described caregiving as physically exhausting due to the combined demands of daily care, including constant movement and supervision. One mother who was a physician caring for an infant explained that caregiving “*causes pain in the bones and muscles*.” Teaching children, particularly older children, was also commonly identified as physically draining when combined with ongoing caregiving responsibilities. The results showed that mothers consistently linked physical exhaustion to disrupted sleep and declining health, particularly among mothers caring for infants, toddlers, or multiple children. Many mothers described ongoing sleep deprivation, often sacrificing rest to complete household tasks, resulting in irregular rest patterns. Such disrupted routines were commonly associated with reported physical symptoms such as back pain, hair loss, and early graying. Mothers with existing health conditions reported greater physical strain due to caregiving responsibilities. A working mother living with celiac disease described how her condition compounded caregiving demands, reporting cramps, constipation, and severe headaches that affected her ability to provide care (Participant 2). For employed mothers, exhaustion and sleep disruption are closely linked to the stress of balancing paid work and caregiving, with many mentioning that rest time in one area is often taken over by demands from the other. These accounts indicate that physical exhaustion was not episodic but cumulative, emerging from the interaction between caregiving intensity, disrupted sleep, and pre-existing health conditions.

#### 3.2.2 Loss of personal time and self-care

Mothers described losing personal time as caregiving occupied most of the day, leaving limited opportunities for rest or self-care. Many mothers found it difficult to engage in activities such as exercise because caregiving schedules coincided with times when children were at home. One participant from the Western region described how caring for a parent who is an older adult alongside her children further restricted her personal time, stating that her mother

“*Constantly wants me by her side… even if there are other people with her, I can't leave to relax or take care of personal matters*.”

Loss of personal time, therefore, limited opportunities for physical recovery and self-care, reinforcing patterns of ongoing fatigue rather than temporary exhaustion.

#### 3.2.3 Positive meaning and fulfillment derived from caregiving

Despite the physical demands of caregiving, mothers across interviews and focus groups emphasized the positive meaning and inner satisfaction derived from caring for their children or parents who are older adults. Emotional closeness and shared daily experiences were described as deeply fulfilling and central to mothers' sense of identity. One mother explained,

“*When I spend quality time with my children and do activities together, I feel happy and fulfilled*” (Focus Group 5).

Other mothers described pride and satisfaction when observing positive behavior or receiving affirming feedback from teachers and others. Participants also framed caregiving in spiritual terms, associating it with gratitude and meaning, and some noted that the positive aspects outweighed the challenges. For many mothers, these sources of meaning appeared to serve as a way to sustain caregiving despite physical strain, rather than eliminating the challenges associated with it.

### 3.3 Theme 3: Gaps between state support and mothers' caregiving needs

This theme reflects the interface between the state and family sectors of the care diamond, examining how mothers experienced misalignment between state-provided services and their caregiving responsibilities. The subthemes are organized using the accessibility, affordability, and quality dimensions applied in international assessments of care service provision (68): (3.1) accessibility of care services; (3.2) affordability and the cost of substituting for public provision; (3.3) quality and mothers' assessments of provision; and (3.4) navigation, workarounds, and compensation strategies. These dimensions are applied across sectors such as education, health, and social care.

#### 3.3.1 Accessibility of care services

Almost all working mothers across the focus groups and interviews highlighted having to navigate gaps in early childcare and workplace-based support, particularly the absence of nurseries in government workplaces such as schools, universities, and hospitals. These gaps required mothers to juggle work and caregiving responsibilities, constraining their ability to balance professional and family roles. One physician explained,

“*I work in a university hospital and have night shifts… It would be easier and more reassuring if there were a nursery at my workplace, where I could keep my children close and feel assured that they are safe while I am working*” (Focus group 3).

In health care, mothers widely acknowledged the availability of public hospitals and primary health care centers, particularly for vaccinations and basic services. However, access and efficiency were recurring concerns, as one mother reported:

“*medical care is available but hard to access easily, even though I am a physician at a university teaching hospital, I do not have fast-track access, and if I want to use business centers, I have to pay out of pocket, as government employees do not have health insurance*” (Focus group 3).

Access was also shaped by eligibility criteria. Mothers described social care services as available but selectively accessible, reporting that they had to manage criteria shaped by income, employment sector, and programme design. Mothers frequently described programmes such as Wusool^1^ and Qurrah^2^ (68, 69) as valuable supports for working mothers, yet many reported being excluded due to income thresholds, despite ongoing caregiving demands:

“*Public services are available, but I'm not eligible for some, such as Qurrah because my salary exceeds the required limit*” (Focus group 6).

Mothers questioned the use of income alone as the sole eligibility criterion for social care programmes, arguing that caregiving intensity, family size, and work demands were overlooked, and calling for broader eligibility criteria. Access was further shaped by location, with mothers in rural areas more likely to depend on public schools due to limited private options.

#### 3.3.2 Affordability and the cost of substituting for public provision

When services were hard to access or seen as insufficient, mothers reported seeking paid options, making affordability a key factor in bridging gaps. Regarding education, most participants reported managing school choice by enrolling their children in private schools, except in the Southern and Northern regions, where public schooling was more common. Mothers described school choice as a key caregiving responsibility, requiring them to compensate for perceived limitations in public education by prioritizing educational quality and children's development. However, choosing private schooling introduced a financial strain that affected caregiving and maternal wellbeing. Despite choosing private schools, many mothers expected the state to play a regulatory role, arguing that families who enroll their children in private education help reduce pressure on public schools and, therefore, warrant protection from rising fees. In rural areas, private options were limited and often unaffordable. In health care, mothers described relying on private hospitals to avoid long waiting times in public facilities, which for some meant paying out of pocket. Participants also described having to compensate for gaps in specialized maternal and postnatal support, which were largely confined to the private sector and often unaffordable. As one mother explained,

“*Psychological and specialized postpartum support is available only through the private sector and is costly*” (Participant 4).

Affordability influenced how service gaps were perceived. Mothers with enough income could turn gaps in access and quality into financial expenses, whereas mothers with fewer resources regarded the same gaps as extra unpaid work and time.

#### 3.3.3 Quality and mothers' assessments of provision

Mothers' judgements about quality shaped whether they used public services at all, and dissatisfaction with public provision was a main reason for moving to the private sector. Concerns about and dissatisfaction with public schooling were particularly pronounced in rural areas. One mother from the Southern region described having to work around deteriorating public school conditions, noting that local schools were old, poorly maintained and characterized by outdated teaching practices. She explained that her children

“*Did not want to go to school because of the way lessons were taught and because the school itself was very old*” (Focus group 5).

Mothers also reported that public nurseries were limited in number and often perceived as inadequate. Mothers often viewed public nurseries as insufficient, citing their limited availability as a reason for choosing private options.

Concerns about adequacy extended to state provisions encountered through employment. Although Saudi labor regulations entitle working women to 12 weeks of fully paid maternity leave, with limited unpaid extensions and additional provisions in cases of infant illness or disability, mothers described these measures as insufficient to support postnatal recovery and early caregiving (70). Mothers also emphasized the need for stronger care-related employment policies, including more flexible work arrangements and longer maternity leave.

#### 3.3.4 Navigation, workarounds, and compensation strategies

Faced with these gaps, mothers described continuous effort to secure care, which formed a substantial and largely invisible part of their caregiving work. Even participants from lower-income groups described strategically navigating between public and private services to secure timely care. One low-income mother from the Southern region reported:

“*Despite having access to a military hospital through my husband, I choose private hospitals in urgent situations to obtain faster care*” (Focus group 5).

Mothers also managed service gaps by working around their employment, taking leave for appointments and follow-ups, especially when their children needed care from several different providers.

Alongside formal programmes, mothers highlighted the importance of supplementing state support with workplace, school-based, and community resources, such as affordable extracurricular activities and community or religious services, such as Qur'an memorisation sessions in mosques. Overall, mothers' perceptions highlight a pattern of ongoing navigation, workarounds, and compensation strategies in response to misalignment between caregiving realities and the design, accessibility, and coordination of existing services. While some mothers could bridge the gaps in access and quality through purchasing due to higher household income, others without such resources took on more unpaid labor and spent extra time to fill those same gaps.

### 3.4 Theme 4: Beyond the state: market and community role in caregiving

In response to gaps in state support identified in theme three, mothers described relying on the market and community sectors of the care diamond to meet their caregiving needs. Market-based services, including private healthcare, childcare, education, and counseling, were the primary sources of support, while community support was limited and informal.

#### 3.4.1 Reliance on market-based care

Access to private care was strongly shaped by insurance coverage, which varied by employment sector. One mother explained that her family's access to private hospitals and postnatal services depended on her husband's employer-provided insurance, whereas others, especially government employees, described paying out of pocket for private services. Private schools and nurseries were similarly described as essential yet costly components of caregiving. Mothers reported relying on private nurseries, while enrolment in private schools was described as a strategic caregiving decision aimed at securing stronger English-language instruction, higher educational standards, and better learning environments. Although these resources were widely viewed as essential and framed as investments in children's development, mothers emphasized that the associated financial costs added to caregiving responsibilities, influencing employment decisions, household budgeting, and contributing to ongoing stress that affected their wellbeing. As one mother pointed out,

“*Private therapeutic and educational sessions with a counselor are very expensive and financially burdensome for parents*” (Focus group 4).

Paid domestic workers represented a significant, often the largest, form of market-based support for most participants. Since their involvement reshaped rather than replaced mothers' caregiving roles, this material is presented in Theme 5 alongside other caregivers.

#### 3.4.2 Community support and its limitations

Alongside paid services, mothers described community support as a potentially valuable yet uneven resource, with informal initiatives easing caregiving for some, while many reported limited or absent support beyond immediate family networks. Many participants stated that “the community doesn't provide much support in caregiving” or that there was “no meaningful community support” available to them (Focus group 2). Several participants highlighted informal community resources such as non-governmental organizations (NGOs), volunteer initiatives, and social media platforms as important supplements to formal services. Mothers described using WhatsApp groups, social media accounts run by parenting experts, and NGO-led workshops to access parenting advice, emotional reassurance, and practical information. As one mother explained,

“*There are social media groups led by parenting experts where mothers learn about childcare and parenting techniques, sometimes for free or at low cost, and occasionally meet at the public library in Dammam to discuss parenting books*” (Focus group 4).

These resources were perceived as helpful in supporting mothers' mental wellbeing by reducing isolation and uncertainty. Participants also expressed a desire for more structured, community-based spaces that could support caregiving and wellbeing. Mothers suggested the need for safe after-school centers and supervised recreational or educational spaces that would engage children in meaningful activities while giving mothers time to rest or attend to personal needs. Overall, non-state care provision through market-based services and informal community support functioned as an essential, but uneven supplement to family care, shifting financial and emotional pressure onto mothers and limiting the capacity of these forms of support to consistently meet caregiving needs and promote family wellbeing.

### 3.5 Theme 5: The role of other caregivers in supporting mothers' caregiving

While state and non-state services shaped caregiving experiences, mothers also described other caregivers as central to organizing caregiving in daily life. Reflecting interactions between the family and market sectors of the care diamond, this theme focuses on two key dimensions of caregiving support: family-based caregiving involving husbands and extended family members, and the role of domestic workers as paid support in daily care. It examined how these forms of support shaped caregiving responsibilities and why they rarely displaced mothers' central coordinating role.

#### 3.5.1 Husbands' involvement in caregiving

Mothers described variation in husbands' involvement, particularly its consistency and practical contribution, as a key factor shaping their caregiving load. Many mothers identified their husbands as important sources of practical support, particularly in transportation, medical appointments, and childcare coordination. As one mother explained:

“*My husband shares all medical and health-related responsibilities equally with me whether it's caring for a sick child, taking them to appointments, or administering medication*” (Participant 1).

When husbands were actively involved, this support enabled mothers to balance caregiving with employment or study. However, husbands' involvement was uneven. Several mothers described limited support due to work demands or prolonged absence, which shifted most caregiving responsibilities onto them. For divorced or separated mothers, the absence of a husband further intensified caregiving, requiring them to manage childcare, finances, and emotional support independently. One divorced mother described fulfilling

“*Both roles,” explaining that her children viewed her as fully responsible, particularly financially, while sympathizing with their father, which added emotional pressure. She also linked this dual responsibility to sleep disruption and depression, stating that she carried “the role of both the woman and the man”* (Participant 3).

These findings illustrate how the absence of spousal support not only increased caregiving tasks but also reconfigured caregiving as an all-encompassing role for mothers.

#### 3.5.2 Extended family as supplementary caregivers

In families where spousal support was limited or unavailable, caregiving responsibilities were not reduced; instead, they were shifted to other family members, such as grandmothers, sisters, or sisters-in-law, who served as supplementary caregivers. Participants described relying on female relatives for childcare, adolescent supervision, and emotional reassurance. One participant highlighted the central role of extended family support in maintaining continuity of caregiving, noting that her mother's involvement was essential to managing family responsibilities during her absences. As she stated,

“*I would not have been able to continue my PhD studies abroad without my mother and family taking care of my children. Leaving them with my family was the only way I could do it*” (Focus group 5).

In contrast, mothers without extended family support emphasized heightened caregiving responsibilities and isolation. One working mother stated:

“*I do everything for my daughter by myself. I live far from my family, I don't have anyone to help me, and I feel isolated with no social life*” (Participant 4).

The support provided by husbands and female relatives varied in scope and duration. Husbands mainly helped with specific daily tasks like transportation, medical appointments, and childcare coordination. Female relatives, on the other hand, often took on ongoing responsibilities for children over longer periods, especially when mothers were away for work or study. In both cases, mothers persisted in viewing themselves as primarily responsible for organizing care.

#### 3.5.3 Domestic workers as paid caregiving support

Domestic workers were described as essential caregiving support for many mothers, particularly in managing household tasks and childcare alongside employment. Domestic workers did not replace mothers' caregiving roles; instead, they reshaped them, as mothers remained ultimately accountable for care even when they delegated tasks. Participants consistently described a reconfiguration of their caregiving role toward oversight and management rather than direct task performance. As one participant noted:

“*Our role shifts from doing the tasks to supervising and coordinating them*” (Focus group 2).

Several mothers explained that domestic help reduced the pressure of housework and allowed them to spend more meaningful time with their children. One interviewee noted that having a domestic worker

“*Lightens the load of washing and cleaning, so I can spend more quality time with my kids*” (Participant 1).

Mothers across both focus groups and interviews described this shift as particularly important when managing long working hours or multiple caregiving responsibilities. Participants also described paid domestic workers as reducing daily caregiving responsibilities by assisting with household tasks and transportation. As one mother stated,

“*Having a driver helps ease my workload, as he takes the children to and from school. When I am busy, the nanny accompanies my children, which makes caregiving much easier for me*” (Focus group 1).

However, the cost of domestic labor was also recognized as an additional financial strain with mothers explaining that reducing these expenses would significantly ease caregiving pressures.

At the same time, reliance on domestic workers raised concerns related to trust, supervision, and dependency. Mothers emphasized that full trust was rarely possible and that continuous monitoring of domestic workers remained necessary:

“*There is no complete trust. I must supervise everything and cannot leave my children for a long time*” (Focus group 3).

Others expressed concern that children might become overly attached to domestic workers. They also noted that mother-child closeness often increased when domestic workers were absent while traveling or on holidays. The presence of domestic workers had mixed effects on marital relationships, with some mothers reporting reduced conflict and others experiencing increased expectations and pressure rather than relief. Overall, this theme demonstrates that while other caregivers played an important role in supporting daily care, their involvement rarely displaced mothers' central responsibility, instead reinforcing caregiving as shared yet predominantly mother-centered work.

### 3.6 Theme 6: Monetary valuation, recognition, and integration of unpaid care within the national economy

This theme builds on the family-sector findings of the care diamond by examining how mothers perceive the economic value and formal recognition of unpaid care. Discussions about the monetary value of unpaid caregiving emerged following the caregiving valuation exercise, including in the interview protocol, in which mothers estimated the time spent on daily caregiving and household tasks. This exercise prompted mothers to reflect on the economic significance of their unpaid caregiving and its absence from formal recognition frameworks.

#### 3.6.1 Valuing unpaid caregiving

The valuation exercise described in the methods produced two sets of estimates. According to mothers' effort-based valuations, the average estimated monetary value of unpaid care work across all activities was 815 SAR per hour (*n* = 21). Considerable variation was observed across participants, reflecting differences in caregiving intensity, access to support, and caregiving contexts, as shown in Table 3. Using the replacement-cost method, the researcher-generated market-based valuation produced a substantially different estimate, highlighting a divergence between mothers' subjective valuations and prevailing market wages for comparable care activities, as shown in Table 4. When replacement-cost hourly wages were summarized across care tasks, the average was 31.8 SAR, and the median was 7.5 SAR. The gap between these two measures reflects the highly uneven market valuation of care work, with professionalized activities such as tutoring commanding substantially higher wages than routine daily care tasks such as childcare, cooking, and cleaning. Reporting both the mean and the median, therefore, provides a more balanced representation of market-based care valuation and clarifies how aggregated figures can obscure variation across care activities.

**Table 3:** Summary of mothers' estimated monetary values of unpaid care work.

Valuation measure	Estimate	Notes
Average	815 SAR/hour	Mothers' perceived effort
Minimum	160 SAR/hour	High support, shared care
Maximum	2,200 SAR/hour	High caregiving demands, restricted employment opportunities due to caregiving responsibilities

Values reflect mothers' own monetary estimates of unpaid care work based on their perceived physical, emotional, and cognitive effort. These estimates are not derived from market wages.

**Table 4:** Replacement-cost–based hourly market wages for unpaid care activities (Saudi Arabia).

Care activity	Comparable paid role	Monthly wage (SAR)	Estimated hourly wage (SAR)
Childcare	Nanny	1,500	6.25
Cooking	Cook	1,800	7.5
Cleaning	Domestic worker	1,500	6.25
Healthcare-related care	Nurse	5,000	14.00
Teaching	Tutor	6,500	125.00

Hourly wages were calculated by dividing reported monthly wages by job-specific assumptions regarding working days and daily working hours, reflecting differences in employment arrangements across care roles (e.g., live-in domestic work, shift-based nursing, and session-based tutoring). These rates were used as a replacement-cost benchmark for comparison with mothers' effort-based valuations of unpaid care work (97, 98).

Across focus groups and interviews, mothers described using familiar market wages as a reference point when estimating the value of their unpaid care work, often referring to local prices for nannies, domestic workers, tutors, and caregivers. However, participants were clear that these comparisons were used only to help express their judgments and did not form the basis of their calculations. Mothers consistently emphasized that the figures they recorded were symbolic and conservative, and did not fully capture the physical, emotional, and cognitive labor involved in caregiving. As one participant explained,

“*The assigned value does not reflect the true effort and emotional burden; my actual effort is much greater*” (Focus group 4).

Importantly, participants' difficulty was not framed as uncertainty about how to value caregiving, but as discomfort with the structural mismatch between the intensity of caregiving labor and its economic representation. Mothers found caregiving emotionally fulfilling, yet unrecognized in economic and policy systems. These reflections did not contradict mothers' sense of satisfaction or commitment to caregiving; rather, they highlighted a perceived gap between the replacement cost of care tasks in the market and the lack of recognition afforded to the same labor when performed within the family. This perceived imbalance between the symbolic monetary valuation and lived caregiving effort reinforced mothers' views that unpaid care work is systematically undervalued despite its central role in sustaining family health and wellbeing.

#### 3.6.2 Recognition and integration of unpaid care within the national economy

Recognition, as mothers used the term, referred to three distinct things: institutional measures, such as the inclusion of caregiving in national planning and social protection systems; policy measures, including flexible employment arrangements, maternity protections, and childcare support; and symbolic measures, such as public acknowledgment of caregiving as essential work rather than a private responsibility. Recognition was not framed solely as direct financial payment. Most participants supported formal recognition in these terms, arguing that it could improve mothers' wellbeing, legitimize caregiving as productive work, and support women's participation in employment or entrepreneurship. Several participants linked such recognition to broader social benefits, including improved family stability and national development. One interviewee stated that

“*Counting unpaid care within the country's economy reflects the value of women's efforts and contributes to productivity*” (Participant 3).

Others suggested that recognition, whether financial, institutional, or symbolic, would motivate mothers to continue providing care and enhance the quality of caregiving. However, views were not uniform. Some mothers were cautious about monetizing caregiving, viewing care primarily as a moral and familial responsibility. Others raised concerns that financial recognition, if poorly regulated, could affect family dynamics or care quality. As one participant cautioned,

“*Recognition would only be appropriate with specific policies and controls, so it is not misused*” (Focus group 3).

Participants often referenced international examples, particularly from other Gulf countries and Singapore, to illustrate alternative approaches to recognizing and supporting unpaid care, including childcare subsidies, direct financial transfers, and comprehensive early childhood support. These examples were used to illustrate what mothers perceived as feasible models for recognizing care while safeguarding quality and fairness. Mothers connected the need for recognition with their experience of caregiving going unseen, describing it as essential work that maintains family wellbeing and allows others to work, yet they remain excluded from the systems that distribute support and entitlements.

Overall, this theme shows that mothers' calls for valuing unpaid care emerged from reflective engagement with their caregiving labor, framing recognition not as a replacement for maternal commitment but as a necessary structural step to address invisibility, inequity, and wellbeing within the care system.

## 4 Discussion

This study explored Saudi mothers' caregiving in relation to family health and wellbeing through focus groups and individual interviews. Six interconnected themes emerged, collectively positioning caregiving as continuous, multidimensional labor shaped by fragmented support systems. The findings reveal that mothers undertake a broad range of daily caregiving activities and responsibilities that extend beyond discrete tasks and carry deep personal meaning, while also entailing significant physical and emotional strains. They also highlight a mismatch between state services and mothers' actual needs, driving reliance on uneven market and community resources, and show that while other caregivers provide support, maternal responsibility remains central. Critically, unpaid care work remains largely unrecognized and undervalued, underscoring the need to integrate it into the national economy. These findings demonstrate that the Saudi care system is stratified: instead of holding a single position in Rogero-García's (38) typology, it shifts between semi-shared and near-unshared arrangements based on households' access to market-based services. This extends Rogero-García's typology beyond its original cross-national application by showing how different care-system configurations can coexist within one country according to household resources (38).

### 4.1 Fragmentation and coordination gaps across the care diamond

Mothers' narratives of navigating disconnected care systems that operate in parallel rather than in coordination reveal not just practical difficulties but also a deeper structural feature of the Saudi care system, which this study situates within Razavi's care diamond framework (29). Prior document analysis identified weak intersectoral coordination between state, market, community and family sectors at the national policy level (30). The present findings extend this by demonstrating the everyday effects of this weak coordination, as mothers must navigate separate care systems that don't communicate with one another, generating an invisible workload that falls disproportionately on mothers as informal care coordinators. State services in health, education, and social protection are available but often hard to access, fragmented across different programmes, and not well-integrated. In response, mothers rely on the market, turning to private schools, private hospitals, and out-of-pocket psychological and postpartum care to fill gaps left by public services. Community support acts as a supplementary resource but is inconsistent and cannot replace formal services. These arrangements are based on a cultural ideal of maternal responsibility for family caregiving, which normalizes depending on mothers to “fill the gaps” caused by fragmented services. It also makes the family sector's role in coordinating work seem natural rather than controversial (71). From the care diamond perspective, the issue isn't missing sectors but weak links between them. The state provides significant formal services; the market offers additional care for those who can access it; and the community provides limited support. However, when coordination across these sectors is weak, it is the family, and within it mothers, who assume responsibility for bridging gaps, as observed in regimes with unstructured intersectoral relationships (12). This study demonstrates that mothers connect public and private healthcare providers, manage referrals, and coordinate school, health, and household activities, including obtaining specialized psychological and disability services. This coordination responsibility increases when formal support fails to reflect the intensity of caregiving. For example, eligibility criteria for programmes such as Qurrah and Wusool may not adequately account for caregiving needs, requiring mothers to undertake additional unpaid coordination work, which further increases their responsibility.

The concentration of care and coordination work in families relates to the concept of familialization, which captures the extent to which care responsibility is retained within families or returned to them rather than redistributed through public or market provision (72). Existing evidence on Saudi Arabia's care systems suggests a distinct variant; services are present, but integration between them is weak, representing a governance gap rather than a resource gap (30). This gap persists due to cultural expectations that caregiving is a private, family-related, and mainly maternal responsibility, which lessens the political pressure to develop formal coordination systems (21, 71). Viewed through the care diamond, this fragmentation is therefore a gendered governance failure rather than a neutral administrative condition, which systematically places responsibility for coordination costs on mothers in the family sector due to weak links between the state, market, and community (12, 29). Describing fragmentation in these terms involves explaining why it counts as a governance failure and how that failure is gendered. Care spans health, education, social protection, and the labor market, requiring coordination across these sectors rather than on provision within any one of them (12). The state's role isn't limited to providing services but includes shaping the entire care system through policies, regulations, or inaction. When this design fails to link sectors, the task of connecting them ultimately falls back on households (29).

This outcome exhibits gendered dimensions in three ways. Firstly, it assumes a household member is available to cover what the system does not provide, with the cultural norm of intensive mothering framing this responsibility as a moral obligation of the mother rather than as labor (35). Secondly, because this labor occurs within the family, it is excluded from formal economic activity and thus remains unrecognized, even as it replaces formal services (15, 21). Thirdly, the ability to buy market alternatives for missing services varies depending on household income, creating socioeconomic inequalities in the caregiving demands experienced by mothers under a single governance structure (20, 29, 30).

### 4.2 Caregiving as multidimensional labor and maternal wellbeing: the role of the mental load

The findings position caregiving as continuous, multidimensional labor that extends beyond physical tasks to include sustained cognitive and emotional work. Mothers described ongoing planning, coordination, and decision-making across children's health, education, and daily routines, highlighting the mental load of caregiving. The mental load is the cognitive labor of anticipating, fulfilling, and monitoring household needs (73). This load is disproportionately shouldered by women, regardless of employment status, consistent with feminist economic scholarship, showing that unpaid care responsibilities remain unevenly distributed along gendered lines (15, 26, 74). Within the care diamond, this finding indicates insufficient support from the state and market sectors; when formal sectors fail to provide enough, the cognitive and logistical tasks of linking fragmented services fall on families, especially mothers (29).

Mental load was evident in participants' responses through the constant coordination of appointments, school activities, and household management, often occurring simultaneously, and at times under significant time pressure. This concentration is heightened by gendered expectations that place mothers as primarily responsible for family care, even when husbands, domestic workers, or formal services are involved. Support tends to minimize specific tasks but does not inherently shift overall responsibility, so mothers remain accountable for anticipating needs, making decisions, and overseeing care. This differed by gender in both scope and duration: husbands' support was limited to specific tasks within daily routines, while female relatives took on ongoing responsibilities during mothers' absences. As a result, redistribution within the family mainly involved women rather than across genders, maintaining care as female labor even when it shifted between households.

The persistence of responsibility even when support is accessible corresponds to what has been referred to as depletion. Depletion is a cumulative erosion of physical and emotional resources that arises not from paid work alone but from the combination of paid employment and unpaid care, especially when there is insufficient support (27). Notably, the findings suggest that depletion also affected formally employed, highly educated mothers who mostly had access to paid domestic help. This finding extends previous evidence from lower-income contexts; although economic advantage and paid domestic help may reduce physical tasks, they do not necessarily lessen mothers' responsibility for care, coordination, or accountability. Consequently, the challenge of balancing paid work and unpaid care continued.

The connection between this combination and mental health is well-documented in the work–family conflict literature. It explains how inter-role conflict occurs when employment and family demands clash. A recent systematic review and meta-analysis of 52 longitudinal studies with 112,714 participants showed that work-to-family conflict predicts later depression, burnout, and general mental distress, regardless of previous mental health, with similar links for family-to-work conflict (75). For working mothers, evidence suggests these effects are influenced by organizational support, flexible work options, and family support, and are intensified by gendered expectations of the “supermom” role and maternal guilt (76). Participants' accounts in this study reflect both aspects: employment responsibilities led to compressed caregiving periods, while caregiving tasks like medical appointments cut into paid work hours. Mothers also reported feeling judged for seeking help, unlike fathers.

These results are especially relevant in Saudi Arabia, where women's workforce participation has increased from around 17% at the start of Vision 2030 to 36.2% in 2024. This surpasses the initial goal of 30% ahead of schedule and has a revised target of 40% by 2030 (77–79). The study indicates that expanding women's employment without improving care infrastructure shifts the associated costs onto mothers. Participants noted that key supports such as workplace nurseries, affordable childcare, flexible work options, and sufficient leave are often missing, limited, or only available to those meeting certain eligibility criteria (80). As a result, higher participation has coincided with increased unpaid care responsibilities, rather than their redistribution. This suggests that sustaining gains in the labor market relies on investing in care systems, not just on women's ability to juggle multiple roles. As shown earlier, care in this context is fragmented and poorly coordinated; consequently, the wellbeing effects discussed here are analyzed as results of these governance structures rather than as a standalone phenomenon. Limited access to childcare, specialist health services, and workplace support, together with rigid employment arrangements, intensified the mental load and contributed directly to physical exhaustion, sleep disruption, and health strain. Maternal wellbeing emerges as shaped not solely by caregiving intensity but by the fit or misfit between caregiving demands and system design. Reducing this misfit depends on institutional design rather than individual coping strategies. It calls for workplace and care policies that address and reduce role conflict at its root, rather than interventions that solely aim to enhance mothers' resilience.

These findings align with public health evidence demonstrating that caregiving responsibilities increases when support is lacking or when institutional structures are misaligned, increasing pressure on mothers managing competing demands (81, 82). Mothers' narratives revealed that emotional fulfillment coexisted with the demands of caregiving. Such duality reflects a well-documented pattern in the caregiving literature, in which emotional rewards function as coping mechanisms rather than indicators of adequate support (71). Overall, by linking the mental load of caregiving to structural conditions, this study reinforces feminist public health arguments that maternal wellbeing is fundamentally shaped by how care is organized, supported, and distributed across society (20, 21, 83).

### 4.3 Reinterpreting the Saudi care system: a market-reliant semi-shared model

Rogero-García's typology classifies care systems as shared, semi-shared, or unshared, depending on how responsibilities are distributed across the state, market, community, and family. In a semi-shared system, the family, mainly women, bears most of the care responsibilities, with minimal support from other sectors (38). Saudi Arabia mostly aligns with this model, in which family-based care, especially by mothers, is central (30). Mothers did not describe the Saudi care system as absent but as inaccessible, unresponsive and poorly coordinated. This is evident in mothers enrolling children in private schools to address gaps in public education, using private hospitals to avoid long waits in public facilities and paying out of pocket for specialized psychological and postpartum care unavailable publicly. Critically, market reliance was not limited to high-income households; low-income mothers also described taking on financial strain to secure timely or specialized care. According to Razavi (84), families bear a personal, gendered, and largely invisible cost when the state plays a residual instead of an integrative role in care provision (29).

Income levels further shape how this semi-shared system operates. Mothers with high incomes or employer insurance can access private services, while lower-income or public-sector employees without medical coverage face limited public services and unaffordable private care. When market access is constrained in this way, care responsibilities fall almost entirely on mothers, resembling an unshared model. This income-based variation reveals a stratified, semi-shared model in which the level of market support and, therefore, the caregiving demands experienced by mothers is shaped by economic status. Rather than confirming a single, fixed semi-shared classification, these findings reveal a flexible, resource-dependent system that shifts between semi-shared and near-unshared arrangements depending on households' market access. This stratification isn't just happenstance; it highlights how market mechanisms are often relied upon instead of public investment. This pattern aligns with broader critiques of residual welfare states, in which the government's retreat from direct services tends to shift caregiving costs onto households least equipped to handle them (20, 21).

This finding extends Rogero-García's typology beyond its initial cross-national context, demonstrating that stratification can also happen within a single nation based on household resources, not just between countries with different welfare systems. It demonstrates that what seems to be a single “semi-shared” regime at the national level can, in reality, produce near-unshared arrangements for low-income mothers, emphasizing the importance of examining care regimes both by welfare-state type and within-country inequalities (38). Viewed through the care diamond, this illustrates that sector boundaries in Saudi Arabia are not fixed but flexible, changing based on household resources. As a result, the same nominal system (semi-shared) can lead to different configurations of state, market, and family involvement for different mothers (29, 38, 84). From a care economy perspective, improving caregiving conditions requires not only expanding public services, which remains crucial, but also reforming the structural factors that make market-based care the default.

### 4.4 Unpaid care invisibility and the recognition gap

The invisibility of unpaid care work reflects a well-documented global structural pattern (6, 85, 86). Unpaid care is systematically excluded from national economic accounts despite its substantial economic value. The International Labor Organization (ILO), estimates that unpaid care work would account for 9% of global GDP, about USD 11 trillion annually (87). Country-level estimates of unpaid care reach 25–37% of GDP in Canada and 61% in the United Kingdom (88, 89). Women and girls perform around 16 billion hours of unpaid care daily, yet this effort is not reflected in official records or policies (90). Within the care diamond, this global pattern indicates a structural failure of the state and market sectors to formally acknowledge work that the family sector, especially mothers, typically absorbs by default (29). In Saudi Arabia, mothers' unpaid caregiving roles are minimally addressed in official frameworks and remain absent from national economic measurements (30). Although the Saudi Vision 2030 raised female labor force participation from 17% in 2016 to 36% in 2024, the conditions rendering caregiving invisible remain unaddressed (91). These include the absence of employer-based insurance for government workers, limited workplace childcare, and the lack of a formal mechanism to recognize or redistribute unpaid care (92). A ministerial decree issued in May 2026 by the Ministry of Human Resources and Social Development (MHRSD), in collaboration with the Ministry of Education (MOE), aims to establish daycare and nursery facilities in public and private workplaces with large numbers of female employees. This indicates an emerging governmental acknowledgment of childcare as vital infrastructure for working mothers, although its implementation remains in the early stages (93, 94). These findings connect this policy gap to mothers' experiences, showing how missing national care policies strain their lives via disrupted work, more time poverty, and ongoing financial and emotional stress.

The valuation exercise conducted highlights the recognition gap from the mothers' perspectives. Mothers' effort-based valuations averaged 815 SAR per hour, as reported in Table 3, substantially higher than the market replacement-cost estimate of 31.8 SAR per hour, derived from the average hourly wage listed in Table 4. This comparison should not be seen as equivalent to national estimates of unpaid care as a percentage of gross domestic product. International estimates and the present exercise operate at different levels and use different units of analysis: national estimates aggregate unpaid care across populations to estimate its economic value, whereas the present exercise compares mothers' own, effort-based assessments of their caregiving with a researcher-calculated replacement-cost estimate based on market wages for comparable paid tasks. Therefore, the figures are not directly comparable. They highlight the same core recognition issue. Replacement-cost methods value caregiving by pricing specific tasks such as cleaning, cooking, childcare, or transportation at current market wages. Mothers' valuations, however, reflected their assessments of the physical, emotional, and cognitive effort, continuous availability, responsibility, planning, mental load, and forgone opportunities involved in caregiving. The gap between these estimates illustrates how market pricing often overlooks key aspects of care that matter most to caregivers. This reflects the broader global recognition gap, in which national economic measures tend to undervalue or ignore unpaid care, despite its crucial role in social reproduction and in supporting the paid economy (15, 20). This difference is important because replacement-cost methods focus on the market price of specific tasks, but they do not consider the ongoing emotional responsibility, mental load, and opportunity costs that mothers consistently identified as central to their caregiving work. This aligns with feminist economic critiques that market measures undervalue care by treating it as interchangeable tasks rather than as ongoing relational labor. This undervaluation is culturally reinforced by the ideology of intensive motherhood, which portrays caregiving as an extension of maternal identity rather than as work that deserves compensation. This framing renders its economic invisibility seem natural, rather than the result of deliberate policy decisions (15).

Valuation estimates varied with support access; mothers with limited support reported higher costs, indicating the recognition gap is greatest among mothers facing the highest caregiving demands. These monetary findings translate directly into specific policy requirements. Mothers' demands encompassed flexible work arrangements, increased childcare support, extended maternity leave, and the formal integration of caregiving into social protection, reflecting a broader shift in care economy scholarship from monetization toward social and institutional recognition of care as a shared societal responsibility (20, 95).

Because coordination gaps and the non-recognition of unpaid care remain unaddressed, these arrangements also have direct impacts on the labor market: mothers in this study limited their employment hours, postponed postgraduate studies, and declined career opportunities due to caregiving demands. These dynamics are consistent with global evidence that unequal care responsibilities constitute a primary structural barrier to women's economic participation (28). In the Saudi context, this creates a policy contradiction: Vision 2030 encourages women's participation in the workforce while only partially addressing the structural conditions that limit it (92). Existing initiatives, such as Qurrah and Wusool programmes, support formally employed mothers but exclude those in precarious, informal, or unpaid caregiving roles. Resolving this contradiction requires policies that recognize unpaid care, redistribute it more equitably, and embed it within employment, social protection, and fiscal planning.

### 4.5 Implications for understanding caregiving arrangements in Saudi Arabia

Taken together, these findings contribute to an understanding of caregiving in Saudi Arabia as a mother-centered and income-stratified care system, shifting between semi-shared and near-unshared arrangements according to households' access to market-based services, and sustained by mothers' continuous labor across family and market sectors, with limited coordination from state and community structures. This study does not define a formal “care economy,” but rather reveals how care is organized and experienced in practice, through mothers' lived realities. This research highlights that prioritizing mothers' voices shows that caregiving sustainability and family health rely not just on individual efforts but also on how care responsibilities are distributed, recognized, and supported across systems. Enhancing maternal wellbeing and aligning care provision with women's expanding social roles are therefore essential for improving maternal wellbeing and family health outcomes.

### 4.6 Study implication

This study emphasizes the role of caregiving as a vital yet often overlooked part of family health and wellbeing in Saudi Arabia. The findings highlight the need for better coordination across different care sectors. Improving integration between state, market, community, and family sectors through clearer service pathways and more consistent eligibility criteria than those currently in place would reduce the responsibility on mothers navigating fragmented systems. Recognizing unpaid care within labor and social policies is equally critical; flexible work arrangements, affordable childcare, and caregiving-inclusive social protection are crucial to supporting maternal wellbeing and enabling more equitable labor market participation. Recent ministerial decisions to extend workplace childcare nurseries in both public and private institutions with large workforces represent an important step in this direction. These actions demonstrate growing policy recognition of childcare as vital infrastructure that enables working mothers and supports the goals of Vision 2030.

### 4.7 Implications for policy and practice

These findings highlight a significant issue in care governance: fragmented and poorly coordinated services across state, market, and community sectors force mothers to act as de facto care coordinators, handling both direct caregiving and unseen coordination tasks. Addressing this governance problem is just as crucial for maternal and family health as expanding the quantity of available services (20, 29).

The key implication is not simply the expansion of individual services but the redesign of care governance through integrated referral systems, harmonized eligibility criteria, investment in care-navigation roles, and formal coordination mechanisms linking health, education, and social care. In the Saudi context, this involves considering care as a fundamental part of social infrastructure within the implementation of Vision 2030: Vision 2030 economic planning units need to explicitly incorporate unpaid caregiving into labor and social policies, including regulations that promote employment sensitive to caregivers, childcare provision, and social protection measures. Existing MHRSD initiatives, like Qurrah and Wusool, show growing recognition of care-related issues. However, wider system-level reforms are needed to reach mothers outside these programs, especially those in informal or unstable jobs. These reforms can help prevent gaps in coordination and childcare provision from limiting women's employment opportunities and putting additional pressure on family wellbeing. Taken together, these directions call for policy measures focused on the recognition and redistribution of unpaid care responsibilities, in line with the “3R” framework for addressing unpaid care work (20), so that efforts to increase women's labor-force participation do not simply intensify mothers' caregiving responsibilities.

### 4.8 Future research

Research on macroeconomic approaches to integrating care into economic models shows that there remains limited empirical evidence on how care is organized, financed, and valued across different national contexts (96). This study contributes micro-level insights into Saudi mothers' valuations of unpaid care and highlights several important directions for future research. First, there is a need for quantitative, nationally representative studies to measure the value of unpaid care through time-use surveys and satellite accounts. Second, policy-modeling studies could explore how different care policies, such as extending public and private childcare, providing caregiver allowances, or pension credits, might affect women's participation in the labor force, income distribution, and macroeconomic outcomes. Third, researchers should conduct longitudinal and mixed-methods studies to monitor how policy changes such as workplace nurseries or childcare subsidies affect mothers' paths in employment, health, and education over time. A dedicated study with Saudi policymakers is essential to understand how they conceptualize the care economy and to identify the assumptions and constraints shaping care-related decision-making. Finally, comparative research across the Gulf and broader Arab countries could examine how various care systems and welfare structures influence the distribution and perception of unpaid caregiving.

### 4.9 Study limitation

The findings are based on mothers' reported experiences and perspectives on caregiving in relation to family health and wellbeing. Although combining focus groups and individual interviews enriched the data, participants in focus groups may have been influenced by others' views when expressing their own. In addition, the study focused exclusively on mothers' perspectives and did not include other caregivers or institutional actors, which may limit the ability to capture the full complexity of care arrangements across sectors.

## 5 Conclusion

This study demonstrates that the main challenge for Saudi mothers is care governance and recognition: services in the state, market, and community sectors function separately instead of as a unified system, and mothers' unpaid care is not acknowledged in economic and policy contexts. As a result, care responsibilities remain concentrated within families and especially fall on mothers, whose continuous, multidimensional caregiving labor absorbs the work of coordinating fragmented support. The findings characterize Saudi Arabia's care arrangements as mother-centered and income-stratified, shifting between semi-shared and near-unshared configurations according to households' access to market-based services. While care is nominally distributed across the state, market, community, and family sectors, its support remains inconsistent and poorly coordinated. Mothers with access to private services, domestic help, or employer-provided insurance can partly compensate for gaps in formal support. In contrast, resource-limited mothers undertake a greater share of unpaid care, which requires more time, effort, and financial sacrifice. As a result, the market functions more as an uneven substitute for coordinated public services, rather than a dependable source of care for all households. This reinforces inequalities and keeps unpaid care invisible and under-recognized, despite its vital role in maintaining family health and wellbeing. These findings indicate that maternal wellbeing depends not only on caregiving demands but also on how care is organized and supported. Addressing these issues requires improving coordination across sectors and incorporating unpaid care in labor and social policy frameworks. In light of Vision 2030, aligning care policy and provision with national development goals is vital to ensure that increased workforce participation does not intensify the demands of unsupported care.

## Data Availability

The raw data supporting the conclusions of this article will be made available by the authors, without undue reservation.

## Footnotes

1 Wusool is a national initiative, provided by the Human Resources Development Fund, that supports employment stability by reducing transportation costs for working women and people with disabilities in the private sector through subsidized rides with licensed ride-hailing companies (68).

2 Qurrah is a national initiative that supports Saudi working women's participation in the labor market by subsidizing childcare costs, with the Human Resources Development Fund covering part of the registration fees at accredited childcare centers through the Qara portal (69).
