# Supplementary material for: Saudi mothers' experiences of caregiving within the care economy: a care diamond analysis
Source: Front Public Health. 2026 Oct 7;14:1897571. doi: 10.3389/fpubh.2026.1897571 (PMC13683006; doi:10.3389/fpubh.2026.1897571)
Supplement: Supplementary file 1 [file Table_1.docx]

**Appendix A: Interview protocol**

**Thematic Sections and Interview Questions**

A. **Daily Caregiving Roles and Responsibilities**
1. Can you walk us through a typical day in your life as a caregiver?

- Prompt: What does your daily routine look like from morning to evening?

2. What caregiving tasks do you perform for your children or other dependents? (such as physical care, medical care, and emotional support)
3. How do your education level and economic situation affect your caregiving responsibilities?
4. Which caregiving tasks do you find most physically or emotionally demanding? Why?
5. Do certain times of day (morning, evening, weekends) feel more stressful or care-intensive than others?

- Prompt: Are there patterns where caregiving becomes overwhelming?

B. **Health and Emotional Well-being Responsibilities**
6. How do you manage your family’s health needs (such as clinic visits, medications, vaccinations)?
7. How do you support your children’s emotional well-being or mental health?

- Prompt: Do you use specific strategies to help them cope with stress, anxiety, or emotional challenges?

8. Are you responsible for caring for any family member with a disability, chronic illness, or special needs?

- If yes, what responsibilities do this involve, and how do they affect your daily life?

C. **Support Systems and Sectoral Interactions (Care Diamond)**
9. Who supports you in your caregiving role?

- Prompt: Can you describe the roles of the following sectors:
  - ***Family members*** (spouse, siblings, in-laws)
  - ***Public services*** (government health clinics, Qurrah or Wusool programs)
  - ***Private sector*** (paid medical care, daycare, pharmacies)
  - ***Community or charity-based programs*** (local NGOs, faith-based support, neighbours, social media groups)

10. Do you feel there is a cultural expectation or stigma associated with seeking external help or support for caregiving?
11. Do you feel this support is sufficient? Where do you experience unmet needs or gaps?

Prompt: Are there situations when no help is available, financial, emotional, or physical?

D. **Financial Value and Unpaid Care Estimation**
12. If you had to hire someone to replace you for your daily caregiving tasks, what would you realistically pay each of these professionals per hour/day/week?

| **Care Activity** | **Comparable Job** | **Hours per Day** | **Hours per Week** | **Wage** | **Weekly Value (SAR)** |
| --- | --- | --- | --- | --- | --- |
| Childcare | Nanny |  |  |  |  |
| Cooking | Cook |  |  |  |  |
| Cleaning | Housemaid |  |  |  |  |
| Healthcare | Nurse |  |  |  |  |
| Tutoring | Teacher |  |  |  |  |
| **Total** |  | | | | |

13. Does the estimated monetary value reflect the actual effort and emotional labour you put into caregiving? Why or why not?
14. Do you believe unpaid caregiving should be counted as part of the national economy?
15. Do you feel your family, community, or government recognises your contributions as a caregiver?

- Follow-up: What would formal recognition mean to you?

E. **The Role of Domestic Workers**
16. Do you currently have a domestic worker (such as a maid or nanny) assisting with care tasks?

- If yes, what caregiving duties does she perform?

17. How does the presence of domestic help affect your caregiving responsibilities?

- Prompt: Does it reduce your burden or shift your role (such as to supervision or coordination)?

18. Do you believe using domestic help changes how caregiving is viewed within your household or society?

- Follow-up: Are there any concerns you have about relying on this form of care?

F. **Personal Impact and Wellbeing**
19. How has your caregiving role affected your physical, emotional, or spiritual well-being both positively and negatively? (such as fatigue, sleep, and nutrition)? (stress, anxiety, isolation)?
20. Are there moments where caregiving brings you satisfaction, pride, or emotional connection? Can you describe one?
21. Has caregiving strengthened any aspects of your social or spiritual life?

Prompt: For example, has it deepened your relationship with your children or parents, or given you a sense of purpose and spiritual fulfilment?

22. Has caregiving influenced your ability to work, study, or pursue personal goals?

- Prompt: What activities have you had to pause or give up?

G. **Recommendations and Reflections**
23. If you could improve one thing in Saudi Arabia to support caregiving mothers better, what would it be?

- Prompt: This could be a policy, a program, a cultural shift, or a financial benefit.

24. What kind of support would make your caregiving responsibilities more manageable or valued?

- Prompt: Think about services, financial recognition, emotional support, or time for self-care.

**بروتوكول مقابلة مجموعة النقاش و المقابلات الفردية Appendix B:**

# **المحاور الموضوعية وأسئلة المقابلة**

# **الأدوار والمسؤوليات اليومية في الرعاية**

1. هل يمكنك أن تصفي لنا يومكِ المعتاد كمقدمة رعاية؟

مُحفّز: كيف يبدو روتينك اليومي من الصباح حتى المساء؟

2. ما المهام التي تقومين بها في تقديم الرعاية لأطفالك أو المعالين الآخرين؟ (مثل الرعاية الجسدية، الرعاية الطبية، الدعم العاطفي)
3. كيف يؤثر مستواك التعليمي و وضعك الاقتصادي على مسؤولياتكِ فيتقديم الرعاية؟
4. ما المهام التي تجدينها أكثر ارهاقاً سواءً جسديًا أو عاطفيًا؟ ولماذا؟
5. هل هناك أوقات معينة من اليوم (الصباح، المساء، عطلة نهاية الأسبوع)) تكون أكثر ضغطًا أو تتطلب رعاية مكثفة؟

مُحفّز: هل تلاحظين أنماطًا يصبح فيها تقديم الرعاية مرهقًا بشكل خاص؟

# **مسؤوليات الصحة والرفاهية العاطفية**

6. كيف تديرين احتياجات أسرتك الصحية؟ (مثل زيارات العيادات، التطعيمات، إعطاء الأدوية،)
7. كيف تدعمين الصحة النفسية و العاطفية لأطفالك؟

مُحفّز: هل تستخدمين استراتيجيات معينة لمساعدتهم على التعامل مع التوتر أو القلق أو التحديات العاطفية؟

8. هل تقدمين الرعاية لأحد أفراد الأسرة من ذوي الإعاقة أو الأمراض المزمنة أو الاحتياجات الخاصة؟

إذا كانت الإجابة نعم، ما هي المسؤوليات التي تتضمنها هذه الرعاية؟ وكيف تؤثر على حياتك اليومية؟

# **أنظمة الدعم والتفاعل القطاعي (مفهوم ماسة الرعاية)**

9. من يدعمكِ في تقديم الرعاية؟

مُحفّز: هل يمكنك وصف أدوار القطاعات التالية؟

- أفراد الأسرة (الزوج، الإخوة، الأصهار او أفراد من عائلة الزوج)
- الخدمات العامة (عيادات الحكومة، برامج قرة أو وصول)
- القطاع الخاص (الرعاية الطبية المدفوعة، الحضانة، الصيدليات)
- المجتمع أو الجمعيات الخيرية (المنظمات غير الربحية، الجيران، مجموعات التواصل الاجتماعي)

10. هل ترين أن هناك توقعًا اجتماعيًا أو نظرة سلبية (وصمة) تجاه الأمهات اللواتي يطلبن الدعم أو المساعدة من خارج الأسرة؟
11. هل تجدين هذا الدعم كافيًا؟ وأين تشعرين بوجود فجوات أو احتياجات غير ملباة؟

مُحفّز: هل هناك مواقف لا يتوفر فيها أي دعم سواءً مالي أو عاطفي أو جسدي؟

# **القيمة المالية وتقدير الرعاية غير مدفوعة الأجر**

12. إذا اضطررتِ لتوظيف شخص للقيام بمهام الرعاية التي تؤدينها يوميًا، ما هو الأجر الواقعي الذي ستدفعينه لكل من هؤلاء المهنيين في الساعة / اليوم / الأسبوع؟
13. هل تعكس القيمة المالية المقدرة الجهد الحقيقي والعبء العاطفي الذي تبذلينه في تقديم الرعاية؟ ولماذا؟
14. هل تعتقدين أن الرعاية غير المدفوعة يجب أن تُحتسب كجزء من الاقتصاد الوطني؟
15. هل تشعرين بأن عائلتك أو مجتمعك أو الحكومة يقدّرون مساهماتكِ كمقدمة رعاية؟

سؤال تابع**: ما الأثر الذي تتوقعينه في حال تم الاعتراف رسميًا بمساهماتك في تقديم الرعاية؟ وماذا يعني لك ذلك شخصيًا؟**

| **أنشطة الرعاية** | **الوظيفة المرادفة** | **عدد الساعات يومياً** | **عدد الساعات أسبوعياً** | **الأجر**  **(ر.س/ ساعة)** | **القيمة الأسبوعية (ر.س)** |
| --- | --- | --- | --- | --- | --- |
| **رعاية الاطفال** | مربية |  |  |  |  |
| **الطبخ** | طباخة |  |  |  |  |
| **التنظيف** | عاملة |  |  |  |  |
| **الدعم الصحي** | ممرضة منزلية |  |  |  |  |
| **الدروس الخصوصية** | معلمة |  |  |  |  |
| **المجموع** |  | | | | |

# **دور العمالة المنزلية**

16. هل لديكِ حاليًا عاملة منزلية (خادمة، مربية) تساعدكِ في مهام الرعاية؟

إذا كانت الإجابة نعم، ما المهام التي تقوم بها؟

17. كيف يؤثر وجود عاملة منزلية على مسؤولياتكِ في تقديم الرعاية؟

مُحفّز: هل يخفف ذلك من الأعباء عليك أو يغير دوركِ في تقديم الرعاية (مثل الإشراف أو التنسيق فقط )؟

18. هل تعتقدين أن وجود عاملة منزلية يغير من نظرة الأسرة أو المجتمع لدور الرعاية؟

سؤال تابع: هل لديكِ أية مخاوف بشأن الاعتماد على هذا النوع من المساعدة؟

# **الأثر الشخصي والرفاهية**

19. كيف أثّر دوركِ كمقدمة رعاية على صحتكِ الجسدية، والعاطفية، والروحية سواء بشكل إيجابي أو سلبي؟ ( ارهاق، اضطرابات نوم، سوء تغذية) (ضغط، قلق، عزلة)
20. هل توجد لحظات تشعرين فيها بالرضا أو الفخر أو الارتباط العاطفي أثناء تقديم الرعاية؟ صِفي لنا واحدة منها.
21. هل ساهم تقديم الرعاية في تعزيز حياتكِ الاجتماعية أو الروحية؟

مُحفّز: مثل تعميق العلاقة مع أطفالكِ أو والديكِ وهل منحتكِ هذه التجربة شعورًا بالرضا الداخلي والقيمة الروحية؟

22. هل أثرت مسؤوليات الرعاية على قدرتكِ على العمل أو الدراسة أو تحقيق أهدافك الشخصية؟

مُحفّز: ما الأنشطة التي اضطررتِ لتأجيلها أو التخلي عنها؟

# **الاقتراحات والانطباعات**

23. إذا كان بإمكانكِ تحسين جانب واحد في السعودية لدعم الأمهات في تقديم الرعاية، ما هو؟

مُحفّز: : قد تكون سياسة معينة، برنامج توعوي، تغيير ثقافي، منفعه مالية.

24. **ما نوع الدعم او اشكال الدعم الذي تعتقدين أنه سيساعدكِ على تحمل مسؤوليات الرعاية بشكل أسهل، أو يجعلكِ تشعرين بأن مجهودكِ مُقدّر؟**

**محفز: فكري في خدمات مثل توفير خدمات مساندة، تعويض مالي، دعم نفسي او اجتماعي، او منح وقت مخصص للاهتمام بنفسك و العناية بصحتك.**

**نموذج الموافقة Appendix C:**

أدوار الأمهات السعوديات في الرعاية وأثرها على صحة الأسرة وجودتها**:** أثير السيف، طالبة دكتوراه، قسم [الصحة العامة، جامعة الإمام عبدالرحمن بن فيصل

تدعو هذه الدراسة الأمهات السعوديات للمشاركة في بحث يهدف إلى استكشاف أدوارهن في الرعاية الأسرية وكيف تؤثر هذه الأدوار على صحة و رفاهية الأسرة.
في حال موافقتك، سيُطلب منك المشاركة في مجموعة نقاش أو مقابلة فردية مدتها تقريبًا 90 دقيقة. سيتم تسجيل الجلسة صوتيًا بعد أخذ موافقتك.

مشاركتك في هذه الدراسة اختيارية تمامًا. لكِ الحق في عدم الإجابة عن أي سؤال، أو إيقاف الجلسة، أو الانسحاب من الدراسة في أي وقت دون الحاجة لذكر الأسباب ودون أي عواقب سلبية عليكِ.

لا توجد مخاطر جسدية متوقعة من المشاركة. قد تتناول بعض الأسئلة موضوعات شخصية أو عاطفية، ويمكنكِ تجاوز أي سؤال لا ترغبين في الإجابة عنه أو إنهاء المشاركة في أي وقت. لن تحصلي على فائدة مباشرة، لكن مشاركتك قد تسهم في تحسين فهم تجارب الأمهات السعوديات في الرعاية الأسرية.

سيتم التعامل مع جميع المعلومات التي تقدمينها بسرية تامة. لن يُذكر اسمك أو أي معلومات تعريفية عنك في أي تقارير أو منشورات بحثية. سيتم حفظ التسجيلات الصوتية والتفريغات النصية في مكان آمن، ولن يطلع عليها إلا فريق البحث. ستُحفظ البيانات لمدة ثلاث سنوات ثم تُتلف بطريقة آمنة.

للاستفسار عن الدراسة، يمكنكِ التواصل مع الباحثة على البريد [الإلكترونيaksaif@iau.edu.sa](mailto:الإلكترونيaksaif@iau.edu.sa)

**الموافقة**
أقرّ بأنني قرأت المعلومات أعلاه وفهمتها، وأُتيحت لي الفرصة لطرح الأسئلة والحصول على الإجابات. أعلم أن مشاركتي طوعية، وأن لي الحق في الانسحاب في أي وقت دون أي تبعات.

☐ أوافق على المشاركة في هذه الدراسة.
☐ أوافق على تسجيل الجلسة صوتيًا لأغراض البحث العلمي.

الاسم _______________________________:
التوقيع أو الموافقة الإلكترونية: ________________________
التاريخ: ____ / ____ / _____

**Appendix D: Consent Form**

**Saudi Mothers’ Caregiving Roles and Their Impact on Family Health and Well‑being**

Atheer AlSaif, PhD candidate, Department of Public Health, Imam Abdulrahman bin Faisal University

You are invited to participate in a research study exploring Saudi mothers’ caregiving roles and their impact on family health and well-being.

If you agree, you will be asked to take part in a focus group discussion or an individual interview lasting about 90 minutes. With your permission, the session will be audio‑recorded.

Participation is completely voluntary. You may choose not to answer any question, and you can stop the discussion or withdraw from the study at any point, without giving a reason and without facing any negative consequences.

There are no expected physical risks. Some questions may feel personal or emotional, but you may skip any question or stop at any time. You will not receive direct personal benefit, but your participation may help improve understanding of Saudi mothers’ caregiving experiences.

Your name and any identifying details will not appear in any reports or publications. Audio recordings and transcripts will be stored securely and accessed only by the research team. Data will be kept for three years and then destroyed.

If you have questions about the study, please contact: [aksaif@iau.edu.sa](mailto:aksaif@iau.edu.sa).

**Consent**

I have read and understood the information above. I have had the opportunity to ask questions. I understand that my participation is voluntary and that I may withdraw at any time without penalty.

☐ I agree to take part in this study.

☐ I agree that the session may be audio‑recorded.

Name: ___________________________

Signature (or electronic consent): ____________________ Date: ____ / ____ / ______

**Appendix E:** Focus group and interviews quotations (English and Arabic)

This table presents original Arabic quotations with their English translations to ensure conceptual equivalence and transparency in qualitative analysis. Quotations are identified by participant or focus group to preserve anonymity.

| **Participant / Focus Group** | **Arabic quotation (original)** | **English quotation (verbatim)** |
| --- | --- | --- |
| Participant 2 | عليّ أن أهيئ نفسي وأُجهز أطفالي في الوقت نفسه. عندما يكون لديك أكثر من طفلين في مدارس مختلفة، عليك الاستيقاظ مبكراً جداً، ويصبح الوضع بشكل عام صعباً. | I have to get myself ready and prepare my children at the same time. When you have more than two children in different schools, you have to wake up very early, and the whole situation becomes difficult to manage |
| Focus group 1 | الجزء الأكثر إرهاقاً هو التنسيق؛ تحديد ما سيُقدم على وجبة الغداء، ومن سيُوصل الأطفال إلى تدريب كرة القدم، وما هي المواد المنزلية التي نحتاج إلى شراءها. قد تبدو هذه الأمور بسيطة، لكن العبء الذهني والتخطيط المستمر مُرهِقان. | The most demanding part is coordination; deciding what’s for lunch, who will take the kids to football practice, and what household items need to be restocked. They seem small, but the mental load and constant planning are draining |
| Focus group 1 | يتم التغاضي عن أي قصور من جانب الأزواج، بينما تتعرض الأمهات للانتقاد لأصغر زلة. | Any shortcomings from husbands are forgiven, while mothers are criticised for the smallest slip |
| Focus group 3 | الأم تتحمل المسؤولية الكاملة | the mother bears full responsibility |
| Focus group 6 | كل ما يتعلق بالصحة في المنزل هو مسؤوليتي | everything related to health at home is my responsibility |
| Participant 3 | الفترات التي تتطلب جهد عاطفي و ذهني أكبر هي عندما يمرض الأطفال، انا بطبيعتي أميل إلى القلق كثيراً. | the periods requiring the greatest emotional and cognitive effort were when children were ill, since I naturally tend to worry a lot |
| Focus group 4 | أضطر إلى أخذ إجازات متكررة من العمل بسبب المتابعة الطبية المستمرة والفحوصات الدورية لطفلي. التنقل بين المستشفيات والأطباء المختلفين يستغرق الكثير من الوقت والجهد، مما يسبب لي صعوبات في العمل. | I have to take regular excuses from work because of the continuous follow-up and medical check-ups for my child. Moving between different hospitals and doctors takes a lot of time and effort and causes me difficulties at work |
| Focus group 2 | لم أتمكن من الدراسة في الخارج لأنني أعطي أولوية لرعاية أطفالي على تقدمي الأكاديمي. | I couldn’t study abroad because I prioritised caring for my children over my academic advancement |
| Focus group 1 | بصفتي أماً متعلمة، أعلم أن الأنشطة اللامنهجية تفيد أطفالي، لذا أحرص على تسجيلهم في الأنشطة الرياضية، ودروس اللغات، ومهارات مثل البرمجة. تنظيم كل هذه الأنشطة يتطلب الكثير من الطاقة والجهد. | As an educated mother, I know extracurricular activities benefit my children, so I make sure to enrol them in sports, language classes, and skills such as coding. Organising all these activities takes a lot of energy and effort |
| Focus group 2 | المستوى التعليمي لا يؤثر على تقديم الرعاية... فالأمر يعتمد على شخصية الأم | The educational level does not make a difference in providing care… it depends on the mother’s personality |
| Focus group 4 | بفضل وضعي المالي الجيد، تمكنت من التسجيل لدى مستشار متخصص في شؤون الأسرة واكتسبت مهارات ساعدتني في رعاية أطفالي - وهي خدمات ما كنت لأتمكن من الحصول عليها لولا دخلي الكافي. | Because of my good financial situation, I was able to enrol with a specialist family consultant and acquire skills that helped me care for my children—services I would not have been able to access without sufficient income |
| Focus group 1 | في الواقع قد يؤدي ارتفاع الوضع الاقتصادي إلى زيادة أعباء الأسرة، حيث ترتفع التوقعات والمطالب، مما يزيد الضغط النفسي على الأمهات. | a higher economic status can actually add to the family’s burdens, as expectations and demands rise, increasing psychological pressure on mothers |
| Focus group 4 | يسبب ألمًا في العظام والعضلات | causes pain in the bones and muscles |
| Participant 2 | تريدني دائماً بجانبها... حتى لو كان هناك أشخاص آخرون معها، لا أستطيع المغادرة للاسترخاء أو الاهتمام بشؤوني الشخصية. | constantly wants me by her side… even if there are other people with her, I can’t leave to relax or take care of personal matters |
| Focus group 1 | عندما أقضي وقتاً ممتعاً مع أطفالي ونقوم بأنشطة معاً، أشعر بالسعادة والرضا. | When I spend quality time with my children and do activities together, I feel happy and fulfilled |
| Focus group 5 | لم يرغب في الذهاب إلى المدرسة بسبب طريقة التدريس ولأن المدرسة كانت قديمة جدًا. | did not want to go to school because of the way lessons were taught and because the school itself was very old |
| Focus group 4 | أعمل في مستشفى جامعي وأعمل في نوبات ليلية... سيكون الأمر أسهل وأكثر طمأنينة لو كانت هناك حضانة في مكان عملي، حيث يمكنني إبقاء أطفالي بالقرب مني فسأشعر بالاطمئنان على سلامتهم أثناء عملي. | I work in a university hospital and have night shifts… It would be easier and more reassuring if there were a nursery at my workplace, where I could keep my children close and feel assured that they are safe while I am working |
| Focus group 2 | تتوفر الرعاية الطبية، لكن يصعب الوصول إليها بسهولة، فعلى الرغم من أنني طبيبة في مستشفى جامعي تعليمي، إلا أنني لا أملك مميزات مثل خدمة المسار السريع، وإذا أردت استخدام مراكز الأعمال، عليّ أن أدفع من جيبي الخاص، لأن موظفي الحكومة لا يملكون تأمينًا صحيًا. | medical care is available but hard to access easily, even though I am a physician at a university teaching hospital, I do not have fast-track access, and if I want to use business centres, I have to pay out of pocket, as government employees do not have health insurance |
| Focus group 5 | على الرغم من إمكانيتي للدخول إلى مستشفى عسكري عن طريق زوجي، إلا أنني أختار المستشفيات الخاصة في الحالات الطارئة للحصول على رعاية أسرع. | Despite having access to a military hospital through my husband, I choose private hospitals in urgent situations to obtain faster care |
| Focus group 6 | تتوفر الخدمات العامة، لكنني لست مؤهلة لبعضها، مثل خدمة برنامج قرة، لأن راتبي يتجاوز الحد المطلوب | public services are available, but I’m not eligible for some, such as Qurrah because my salary exceeds the required limit |
| Participant 4 | الدعم النفسي والرعاية المخصصة لما بعد الولادة غير متوفرة إلا من خلال القطاع الخاص وهو مكلف. | Psychological and specialised postpartum support is available only through the private sector and is costly |
| Focus group 4 | تُعد الجلسات العلاجية والتدريبية مع مستشار خاص مكلفة للغاية وتُشكل عبئًا ماليًا على الآباء. | private therapeutic and educational sessions with a counsellor are very expensive and financially burdensome for parents |
| Focus group 1 | وجود سائق يُخفف عني عبء العمل و الرعاية، فهو يُوصل الأطفال من وإلى المدرسة. وعندما أكون مشغولة، تُرافق الخادمة أطفالي، مما يُسهّل عليّ رعايتهم كثيرًا. | Having a driver helps ease my workload, as he takes the children to and from school. When I am busy, the housemaid accompanies my children, which makes caregiving much easier for me |
| Focus group 2 | المجتمع لا يقدم الكثير من الدعم في مجال الرعاية | the community doesn’t provide much support in caregiving |
| Focus group 2 | لا يوجد دعم مجتمعي نافع | no meaningful community support |
| Focus group 4 | توجد مجموعات على وسائل التواصل الاجتماعي يديرها خبراء في مجال التربية، حيث يعلمون الأمهات عن رعاية الأطفال وأساليب التربية، أحيانًا تكون الجلسات مجانًا أو بتكلفة منخفضة، وتجتمع هذه المجموعات أحيانًا في المكتبة العامة في الدمام لمناقشة كتب التربية. | There are social media groups led by parenting experts where mothers learn about childcare and parenting techniques, sometimes for free or at low cost, and occasionally meet at the public library in Dammam to discuss parenting books |
| Participant 1 | يتقاسم زوجي معي جميع المسؤوليات الطبية والصحية بالتساوي - سواء كان ذلك رعاية طفل مريض، أو اصطحابه إلى المواعيد الطبية، أو إعطاء الدواء. | My husband shares all medical and health-related responsibilities equally with me—whether it’s caring for a sick child, taking them to appointments, or administering medication |
| Participant 3 | دور المرأة والرجل معاً. | the role of both the woman and the man |
| Focus group 5 | لم أكن لأتمكن من مواصلة دراستي للدكتوراه في الخارج لولا رعاية والدتي وعائلتي لأطفالي. كان تركهم مع عائلتي هي الطريقة الوحيدة المتاحة لي. | I would not have been able to continue my PhD studies abroad without my mother and family taking care of my children. Leaving them with my family was the only way I could do it |
| Participant 4 | أقوم برعاية ابنتي كاملةً بمفردي. أعيش بعيدًا عن عائلتي، وليس لديّ من يساعدني، وأشعر بالعزلة التامة وانعدام الحياة الاجتماعية. | I do everything for my daughter by myself. I live far from my family, I don’t have anyone to help me, and I feel isolated with no social life |
| Focus group 2 | تحول دورنا من القيام بالمهام إلى الإشراف عليها وتنسيقها. | Our role shifts from doing the tasks to supervising and coordinating them |
| Participant 1 | يخفف من عبء الغسيل والتنظيف، مما يسمح لي بقضاء المزيد من الوقت الممتع مع أطفالي. | lightens the load of washing and cleaning, so I can spend more quality time with my kids |
| Focus group 3 | لا توجد ثقة كاملة. يجب عليّ الإشراف على كل شيء ولا يمكنني ترك أطفالي مع العاملة المنزلية لفترة طويلة. | There is no complete trust. I must supervise everything and cannot leave my children for a long time |
| Focus group 4 | القيمة المقدرة لا تعكس الجهد الحقيقي والعبء العاطفي - جهدي الفعلي أكبر بكثير | The assigned value does not reflect the true effort and emotional burden—my actual effort is much greater |
| Participant 3 | إن احتساب الرعاية غير مدفوعة الأجر ضمن اقتصاد الدولة يعكس قيمة جهود المرأة ويساهم في الإنتاجية. | counting unpaid care within the country’s economy reflects the value of women’s efforts and contributes to productivity |
| Focus group 3 | الاعتراف بالرعاية غير مدفوعة الأجر يكون مناسبًا فقط في ظل وجود سياسات وضوابط محددة، حتى لا يُساء استخدامه. | recognition would only be appropriate with specific policies and controls, so it is not misused |
